# Protective roles of MITOL against myocardial senescence and ischemic injury partly via Drp1 regulation

**DOI:** 10.1016/j.isci.2022.104582

**Published:** 2022-06-11

**Authors:** Takeshi Tokuyama, Hideki Uosaki, Ayumu Sugiura, Gen Nishitai, Keisuke Takeda, Shun Nagashima, Isshin Shiiba, Naoki Ito, Taku Amo, Satoshi Mohri, Akiyuki Nishimura, Motohiro Nishida, Ayumu Konno, Hirokazu Hirai, Satoshi Ishido, Takahiro Yoshizawa, Takayuki Shindo, Shingo Takada, Shintaro Kinugawa, Ryoko Inatome, Shigeru Yanagi

**Affiliations:** 1 Laboratory of Molecular Biochemistry, School of Life Sciences, Tokyo University of Pharmacy and Life Sciences, Hachioji, Tokyo, Japan; 2 Division of Regenerative Medicine, Center for Molecular Medicine, Jichi Medical University, Tochigi, Japan; 3 Diagnostics and Therapeutics of Intractable Diseases, Intractable Disease Research Center, Graduate School of Medicine, Juntendo University, Tokyo, Japan; 4 Laboratory of Molecular Biochemistry, Department of Life Science, Faculty of Science, Gakushuin University, Mejiro, Tokyo, Japan; 5 Department of Applied Chemistry, National Defense Academy, Yokosuka, Japan; 6 First Department of Physiology, Kawasaki Medical School, Kurashiki, Japan; 7 Graduate School of Pharmaceutical Sciences, Kyushu University, Fukuoka, Japan; 8 National Institute for Physiological Sciences, National Institutes of Natural Sciences, Okazaki, Japan; 9 Department of Neurophysiology and Neural Repair, Gunma University Graduate School of Medicine, Maebashi, Gunma, Japan; 10 Department of Microbiology, Hyogo College of Medicine, Nishinomiya, Japan; 11 Research Center for Advanced Science and Technology, Shinshu University, Matsumoto, Nagano, Japan; 12 Department of Cardiovascular Research, Shinshu University School of Medicine, Matsumoto, Nagano, Japan; 13 Department of Life Innovation, Institute for Biomedical Sciences, Interdisciplinary Cluster for Cutting Edge Research, Shinshu University, Matsumoto, Nagano, Japan; 14 Department of Lifelong Sport, School of Sports Education, Hokusho University, Ebetsu, Japan; 15 Department of Cardiovascular Medicine, Faculty of Medical Sciences, Kyushu University, Fukuoka, Japan; 16 Division of Cardiovascular Medicine, Research Institute of Angiocardiology, Faculty of Medical Sciences, Kyushu University, Fukuoka, Japan

**Keywords:** Physiology, Cellular physiology, Molecular biology, Developmental biology

## Abstract

Abnormal mitochondrial fragmentation by dynamin-related protein1 (Drp1) is associated with the progression of aging-associated heart diseases, including heart failure and myocardial infarction (MI). Here, we report a protective role of outer mitochondrial membrane (OMM)-localized E3 ubiquitin ligase MITOL/MARCH5 against cardiac senescence and MI, partly through Drp1 clearance by OMM-associated degradation (OMMAD). Persistent Drp1 accumulation in cardiomyocyte-specific MITOL conditional-knockout mice induced mitochondrial fragmentation and dysfunction, including reduced ATP production and increased ROS generation, ultimately leading to myocardial senescence and chronic heart failure. Furthermore, ischemic stress-induced acute downregulation of MITOL, which permitted mitochondrial accumulation of Drp1, resulted in mitochondrial fragmentation. Adeno-associated virus-mediated delivery of the MITOL gene to cardiomyocytes ameliorated cardiac dysfunction induced by MI. Our findings suggest that OMMAD activation by MITOL can be a therapeutic target for aging-associated heart diseases, including heart failure and MI.

## Highlights

• MITOL is essential for maintaining cardiac function partly via Drp1 clearance
• MITOL deficiency causes cardiac aging partly via Drp1 accumulation
• Ischemic stress induces a rapid downregulation of MITOL
• MITOL expression attenuates cardiac dysfunction in acute myocardial infarction

## Graphical abstract

**Figure:**
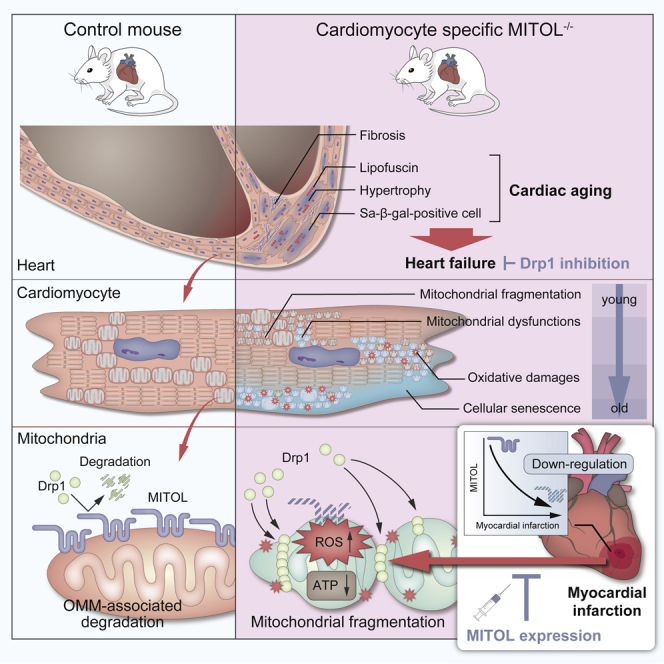

## Introduction

Mitochondria are responsible for cellular metabolism, creating energy, and maintaining calcium homeostasis according to their own dynamic morphological changes that continuously undergo fusion and fission (Dimmer and Scorrano, 2006; Friedman and Nunnari, 2014; Mishra and Chan, 2014). Mitochondrial dysfunction is associated with various unwanted effects, including cytotoxic oxygen radicals and reduced ATP production; both contribute to senescence (Wallace, 2005). Because mitochondrial morphology and functions are tightly associated with each other (Frank et al., 2001; Youle and Karbowski, 2005), mitochondrial fission is pathologically associated with the release of cytochrome c from mitochondria that activates apoptosis signaling (Youle and van der Bliek, 2012) and may contribute to postischemic heart damage (Ong et al., 2010). After MI, the heart is characterized by mitochondrial dysfunction. The relevance of mitochondrial abnormalities in chronic heart failure progression is broadly accepted (Heather et al., 2010; Hori and Nishida, 2009). Mounting evidence demonstrates that mitochondrial fragmentation is closely implicated in this pathological progress, suggesting that unveiling the mechanisms of mitochondrial network regulation will inform an additional therapeutic strategy for heart failure.

Mitochondrial fission is specifically mediated by the dynamin-related guanosine triphosphatase (GTPase) protein 1 (Drp1), which accumulates from the cytosol to the OMM (Hoppins et al., 2007). Four membrane proteins of the OMM—mitochondrial fission protein 1 (Fis1), mitochondrial fission factor (Mff), and mitochondrial dynamics proteins of 49 and 51 kDa (MiD49 and MiD51)—have been proposed as receptors that recruit Drp1 to mitochondria (Loson et al., 2013). Accumulated Drp1 oligomerizes and encircles a mitochondrion to constrict the mitochondrial membrane (Kalia et al., 2018). For Drp1 activity, translocated Drp1 is regulated by several different posttranslational modifications, including phosphorylation (Chang et al., 2013), SUMOylation (Figueroa-Romero et al., 2009), S-nitrosylation (Cho et al., 2009), polysulfidation (Akaike et al., 2017), and O-GlcNAcylation (Gawlowski et al., 2012). In addition, it has been reported that the ubiquitination of Drp1 by mitochondrial ubiquitin ligase MITOL (also referred to as MARCH5) regulates the amount of mitochondrial Drp1 (Nakamura et al., 2006; Yonashiro et al., 2006), and plays a role in the control of mitochondrial morphology and functions.

It has been reported that MITOL regulates various cellular targets and pathways, including the mitochondria-endoplasmic reticulum interaction (Sugiura et al., 2013), removal of misfolded proteins (Sugiura et al., 2011; Yonashiro et al., 2009), maintenance of stemness (Gu et al., 2015), control of innate immunity (Yoo et al., 2015), and mitophagy (Chen et al., 2017; Koyano et al., 2019). The relatively broad functions of MITOL pathways suggest a critical role for MITOL in maintaining mitochondrial homeostasis. Regulation of mitochondrial morphology appears to be the most well-investigated function of MITOL (Cherok et al., 2017; Karbowski et al., 2007; Wang et al., 2016; Xu et al., 2016; Zhang et al., 2016). In addition, it has been reported that MITOL interacts with mitochondrial pro-fission factors such as Fis1, MFF, and Mid49. Even with these insights, the effects of MITOL-dependent ubiquitylation of fission factor Drp1 on mitochondrial dynamics remain unclear. Consequently, the mechanisms of MITOL action, their physiological role, and how they are regulated warrant further study.

MITOL is also connected to ubiquitin-mediated degradation of mitochondrial proteins during OMM-associated degradation (OMMAD) (Xu et al., 2016). OMMAD is a protein removal system on the outer mitochondrial membrane by the ubiquitin-proteasome system (UPS). Therefore, OMMAD serves as a guardian of mitochondria from cytoplasmic toxicity factors. Notably, it has been reported that target proteins of OMMAD regulate apoptosis or mitochondrial membrane dynamics (Karbowski and Youle, 2011). Excess regulatory molecules frequently accumulate on dysfunctional mitochondria, such as the Drp1-dependent mitochondrial fragmentation observed in heart diseases. For this reason, understanding OMMAD is important to restore mitochondrial function. Yet, the clearance system of mitochondrial Drp1 is still not well understood.

In this study, we characterized cardiomyocyte-specific MITOL conditional-knockout mice, which exhibited systolic dysfunction with intense fibrosis from six months after MITOL deletion, and all died within one year. Notably, aging symptoms, such as β-galactosidase activity and lipofuscin pigmentation, preceded these events. Because MITOL expression is downregulated in various aged mouse tissues (including in aging hearts), MITOL is a critical regulatory factor for heart aging and aging-associated heart failure. Finally, we propose that OMMAD activation by MITOL can be a therapeutic target for MI.

## Results

### MITOL suppresses cellular senescence partly via Drp1 degradation

**Figure 1:**
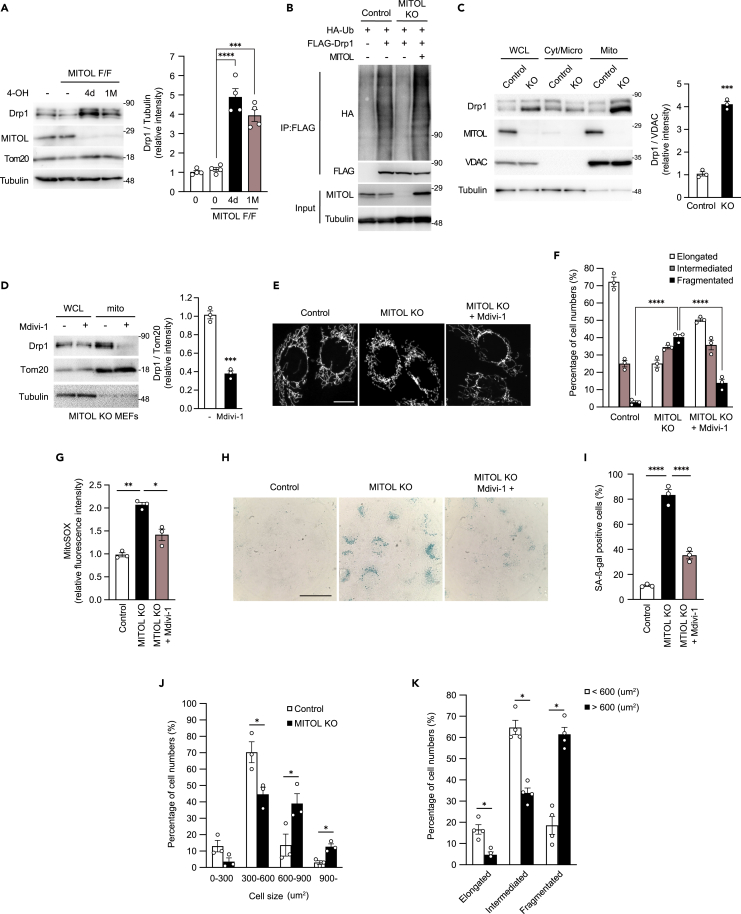
MITOL knockout induces the Drp1-dependent fragmentation of mitochondria, followed by the vulnerability and senescence in MEFs (A) Accumulation of Drp1 in MITOL knockout (MITOL-KO) MEFs. Cre-ERT2-expressing WT MITOL (WT) or MITOL^flox/flox^ MEFs were treated with 0.8 μM 4-hydroxytamoxifen (4-OHT) for one day. Four days after 4-OHT treatment was defined as 4d, and more than one month after 4-OHT treatment was defined as 1 M. Knockout of MITOL was confirmed and Drp1 expression was detected by immunoblot (IB) analysis with anti-MITOL and anti-Drp1 antibodies, respectively. Error bars represent ±SEM (n = 4). Analysis was performed with one-way ANOVA followed by Bonferroni *post hoc* analysis. ∗∗∗p < 0.001, ∗∗∗∗p < 0.0001. (B) MITOL ubiquitinates Drp1. MITOL^F/F^ MEFs (control) or MITOL-KO MEFs were co-transfected with/without indicated expression vectors for FLAG-tagged Drp1, HA-tagged ubiquitin and non-tagged MITOL for 24 h and lysates were immunoprecipitated (IP) with anti-FLAG antibody, followed by immunoblotting with anti-HA antibody or anti-FLAG antibody. Cells were treated with MG132 (10 μM) for 10 h before harvest. Whole lysates were immunoblotted with anti-MITOL and anti-tubulin antibodies. (C) Accumulation of Drp1 in mitochondrial fraction. IB assay was performed on lysates of whole (WCL), cytosolic (Cyt/Micro), and mitochondrial (mito) fractions isolated from MITOL^F/F^ MEFs (control) or MITOL-KO (KO) MEFs with Drp1 antibody. Anti-VDAC and anti-tubulin antibodies were used as a mitochondrial marker and a cytosolic marker, respectively. Mitochondrial Drp1 was normalized by the intensity of VDAC. Error bars represent ±SEM (n = 3). ∗∗∗p < 0.001 (Student’s *t*-test). (D) Mdivi-1 inhibits Drp1 accumulation in MITOL-KO mitochondria. MITOL-KO MEFs were treated with or without 5 μM Mdivi-1 for 12 h and lysates of whole and mitochondrial fractions were immunoblotted with anti-Drp1 antibody. Anti-Tom20 and anti-tubulin antibodies were used as a mitochondrial marker and a cytosolic marker, respectively. Mitochondrial Drp1 was normalized by the intensity of Tom20. Error bars represent ±SEM (n = 3). ∗∗∗p < 0.001 (Student’s t-test). (E and F) Mdivi-1 attenuates mitochondrial fragmentation in MITOL-KO MEFs. MITOL^F/F^ MEFs (control), MITOL-KO MEFs (MITOL KO), and MITOL-KO MEFs treated with 5 μM Mdivi-1 for 12 h (MITOL KO + Mdivi-1) were stained with MitoTracker Green and mitochondrial morphologies were compared. Bar, 10 μm (E). Percentages of cells showing each mitochondrial morphology were calculated from 100 cells of each MEFs shown in E. Mean ± SEM (n = 3). Analysis was performed with two-way ANOVA followed by Bonferroni *post hoc* analysis. ∗∗∗∗p < 0.0001 (F). (G) Mdivi-1 attenuates mitochondrial ROS production in MITOL-KO MEFs. MITOL^flox/flox^ MEFs (control) or MITOL-KO (KO) MEFs treated with or without 5 μM Mdivi-1 for 12 h were stained with MitoSOX and mitochondria-derived superoxide generation was measured by flow cytometric analysis. Bar graphs show relative levels of mean fluorescence intensity of MitoSOX compared with that of MITOL^flox/flox^ MEFs (control). Mean ± SEM (n = 3). Analysis was performed with one-way ANOVA followed by Bonferroni *post hoc* analysis. ∗p < 0.05, ∗∗p < 0.01. (H and I) Accumulation of senescent cells in MITOL knockout MEFs was restored by Mdivi-1 treatment. Cytochemical staining of SA-β-gal activity in MITOL^flox/flox^ MEFs (control), or MITOL-KO MEFs treated with or without 5 μM Mdivi-1 for six hours. Bar, 50 μm (H). The bar graph shows the percentages of SA-β-gal positive cells (100 cells/experiment, n = 3). Mean ± SEM. Analysis was performed with one-way ANOVA followed by Bonferroni *post hoc* analysis. ∗∗∗∗p < 0.0001 (I). (J) MITOL-KO MEFs promote age-related hypertrophy. The areas of MITOL^flox/flox^ MEFs (control) and MITOL-KO MEFs were measured. Percentages of cells showing each cell size were calculated from 100 cells of each MEFs. Mean ± SEM (n = 3). ∗p < 0.05. (K) Mitochondrial fragmentation correlates with age-related hypertrophy in MITOL-KO MEFs. MITOL-KO MEFs were stained by anti-Tom20 and anti-Actin antibodies. Percentages of cells showing each mitochondrial morphology were calculated from 100 cells of each MEFs. Mean ± SEM (n = 4). ∗p < 0.05.

Our previous study showed that MITOL ubiquitinates and degrades Drp1, a mitochondrial fission factor (Yonashiro et al., 2006). However, the detailed mechanism of Drp1 degradation is not well understood. Here, we investigated the detailed mechanism of MITOL-induced Drp1 degradation and the long-term effects of MITOL deficiency using mouse-derived embryonic fibroblasts (MEFs). We generated tamoxifen-inducible MITOL-knockout MEFs immortalized by SV40 large T antigen (Sugiura et al., 2013). Drp1 was accumulated in MITOL^flox/flox^ (MITOL^F/F^) (hereafter referred to as MITOL-KO) MEFs four days or more than one month after 4-hydroxytamoxifen (4-OHT) treatment compared to WT or tamoxifen-untreated MITOL^F/F^ MEFs (Figure 1A). To confirm MITOL-dependent Drp1 ubiquitination, we transfected Drp1 with or without MITOL to MITOL-KO MEFs and found that Drp1 was ubiquitinated only after the transfection of both Drp1 and MITOL (Figure 1B). Furthermore, a high accumulation of Drp1 was observed in the mitochondrial-rich fraction of MITOL-KO MEFs (Figure 1C).

To assess the toxicity of Drp1 in MITOL-KO MEFs, we used a Drp1 inhibitor—Mdivi-1—or siRNA targeting Drp1 to complement the effect of Mdivi-1 (Cassidy-Stone et al., 2008). We first confirmed that Mdivi-1 blocked the accumulation of Drp1 to mitochondria in MITOL-KO MEFs (Figure 1D). The introduction of siRNA targeting Drp1 suppressed the expression of endogenous Drp1 by 80% compared with that of scramble siRNA (Figure S1A). Mitochondrial fragmentation was induced in MITOL-KO MEFs and restored by Mdivi-1 treatment (Figures 1E and 1F) or siRNA transfection (Figures S1B and S1C). Mitochondrial fragmentation is often associated with enhanced reactive oxygen species (ROS) production (Kluge et al., 2013; Shenouda et al., 2011). In line with the accumulation of Drp1 to mitochondria, mitochondrial ROS production was enhanced in MITOL-KO MEFs and was restored by Mdivi-1 treatment (Figure 1G) or siRNA (Figure S1D). These data suggest that ROS production in MITOL-KO MEFs was induced, at least in part, by Drp1 accumulation because of the OMMAD abnormalities.

It is widely reported that intracellular ROS accelerates cellular senescence (Miquel et al., 1980). We, therefore, monitored cellular senescence by β-gal staining. β-gal positive cells accumulated in MITOL-KO MEFs, whereas these cells decreased following Mdivi-1 treatment (Figures 1H and 1I) or siRNA targeting Drp1 (Figures S1E and S1F). These data indicate that cellular senescence observed in MITOL-KO MEFs was mainly the cause of Drp1 toxicity. Another known phenotype of senescent cells is cellular hypertrophy. As expected, cellular hypertrophy was induced by MITOL knockout (Figure 1J). In addition, cellular hypertrophy was highly correlated with the fragmentation of mitochondria in MITOL-KO MEFs (Figure 1K), suggesting mitochondrial fission-dependent cellular hypertrophy. These findings indicate that Drp1 hyper-accumulation because of the OMMAD dysfunction on mitochondria caused by MITOL knockout leads to the production of ROS and cellular senescence.

### MITOL is a prerequisite for cardiac mitochondrial morphology and function

**Figure 2:**
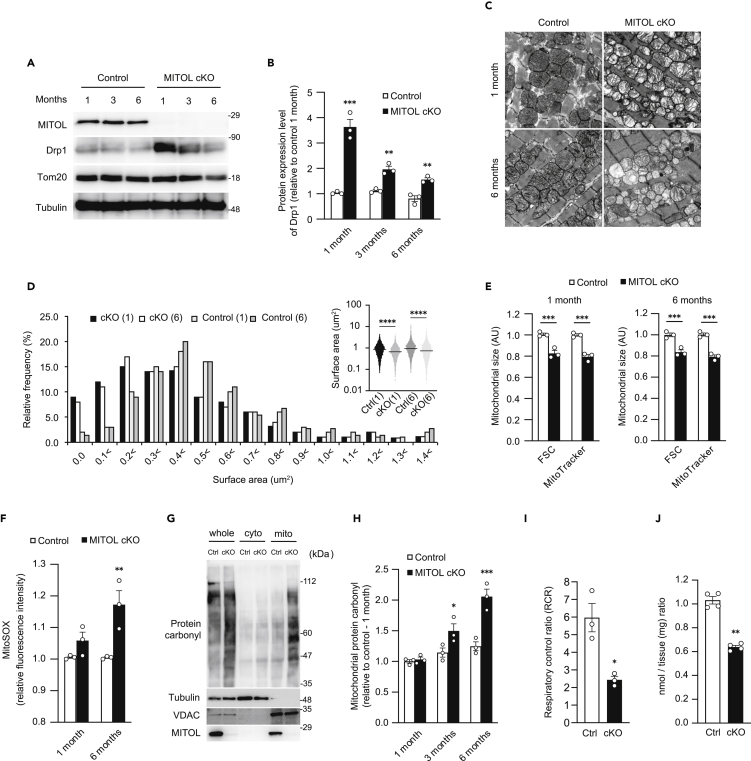
Mitochondrial morphological change by accumulated Drp1 and dysfunction in heart-specific MITOL-KO mice (A and B) Heart-specific MITOL KO enhances Drp1 accumulation in the heart. Samples were collected from the hearts of MITOL^flox/flox^ (Control) and MITOL^flox/flox^;αMHC-Cre^Mer^ (MITOL cKO) mice treated with tamoxifen for the indicated periods, followed by immunoblotting with indicated antibodies. The relative protein levels of Drp1 were quantified by densitometry. Data are standardized to tubulin levels and are expressed relative to control mice. Mean ±SEM (n = 3). Analysis was performed with two-way ANOVA followed by Bonferroni *post hoc* analysis. ∗∗∗p < 0.001, ∗∗p < 0.01. (C and D) Abnormal morphologies of mitochondria in MITOL-KO mouse cardiomyocytes. Representative electron microscopic images of mitochondria from the hearts of MITOL^flox/flox^ (Control) and MITOL^flox/flox^;αMHC-Cre^Mer^ (MITOL cKO) mice treated with tamoxifen for the indicated periods. 27,600-fold magnification (C). Mitochondrial size was represented as median surface area, and frequency distributions of mitochondrial surface were calculated from mitochondria imaged by TEM (D). Kruskal-Wallis test, ∗∗∗∗p < 0.0001. Data are median values. Bar, 500 nm. (E) Decreased size of mitochondria in MITOL-KO mouse cardiomyocytes. Cardiac mitochondrial areas of MITOL^flox/flox^ (Control), and MITOL^flox/flox^;αMHC-Cre^Mer^ (MITOL cKO) mice treated with tamoxifen for the indicated periods were measured. Mitochondrial fractions isolated from cardiomyocytes of MITOL^flox/flox^ and MITOL^flox/flox^;αMHC-Cre^Mer^ mice treated with tamoxifen for the indicated periods were stained with MitoTracker, followed by flow cytometric analysis. Bar graphs show the relative levels of mean fluorescence intensity of forward-scatter (FSC) and MitoTracker to MITOL^flox/flox^ mice. Mean ±SEM (n = 3). ∗∗∗p < 0.001. (F) Mitochondrial ROS was upregulated in MITOL-KO mouse cardiomyocytes. Cardiac mitochondrial fractions of MITOL^flox/flox^ (Control), and MITOL^flox/flox^;αMHC-Cre^Mer^ (MITOL cKO) mice treated with tamoxifen for the indicated periods were stained with MitoSOX and mitochondrial-derived superoxide generation was measured by flow cytometric analysis. Bar graph shows the relative levels of mean fluorescence intensity of MitoSOX to MITOL^flox/flox^ mice. Mean ± SEM (n = 3). Analysis was performed with two-way ANOVA followed by Bonferroni *post hoc* analysis. ∗∗p < 0.01. (G and H) MITOL knockout induces mitochondrial oxidative damage in cardiomyocytes. Protein carbonyl contents of cardiac mitochondrial extracts were determined by protein carbonyls western blot detection kit (G). The levels of carbonylated proteins were quantified by densitometry. Data are standardized to VDAC levels and are expressed relative to MITOL^flox/flox^ (Ctrl) mice prior to tamoxifen treatment. Mean ±SEM (n = 3). Analysis was performed with two-way ANOVA followed by Bonferroni *post hoc* analysis. ∗p < 0.05, ∗∗∗p < 0.001. (H). (I) Reduced oxygen consumption rate of mitochondria isolated from MITOL-KO mouse cardiomyocytes. The respiratory control ratio (RCR) represents the mitochondrial coupling state. Mean ±SEM (n = 3). ∗p < 0.05, Student’s t-test. (J) Reduced ATP content in the MITOL-KO heart. ATP content was measured by luciferase assay. Mean ±SEM (n = 4). ∗∗p < 0.01, Student’s *t*-test.

Cardiomyocytes require high levels of ATP for pulsation (Dorn, 2013). Mitochondrial morphological changes are often seen in human heart diseases, and knockout mice of mitochondrial fusion factor Mfn1/2 cause cardiac abnormalities (Chaanine et al., 2019; Chen and Knowlton, 2011; Dorn, 2015), suggesting that maintenance of mitochondrial morphology (dynamics) plays an essential role in maintaining cardiac function. We examined whether MITOL is involved in the maintenance of cardiac function by maintaining the normal function of myocardial mitochondria. To prove the involvement of MITOL in the regulation of cardiomyocytes, we crossed MITOL^F/F^ mice with tamoxifen-inducible, α-myosin heavy-chain (αMHC) Cre-transgenic mice to generate tamoxifen-inducible (Sohal et al., 2001), heart-specific MITOL-KO mice-termed MITOL-cKO mice (Figures S2A–S2C). MITOL in cardiomyocytes disappeared one month post-tamoxifen treatment; the accumulation of Drp1 was observed during the same period (Figures 2A and 2B). Drp1 accumulation significantly decreased ∼six months after MITOL knockout, suggesting that unknown Drp1 downregulation mechanisms exist *in vivo*. Because MITOL has several mitochondrial substrates (Cherok et al., 2017; Karbowski et al., 2007; Xu et al., 2016; Zhang et al., 2016), we confirmed the accumulation of mitochondrial proteins in knockout mice. Among the proteins regulating mitochondria morphology, Drp1 accumulated most on mitochondria and, Mid49 was also accumulated in the MITOL-cKO heart. Surprisingly, no significant accumulation of Fis1 and MFF was observed (Figure S2D). A phosphorylated form of Drp1, the pro-fission modification, was also observed in the MITOL-cKO heart (Figure S2E).

Electron microscopy and flow cytometric analysis of mitochondrial size revealed smaller mitochondria with abnormal cristae structure in the absence of MITOL, which suggested impaired mitochondrial dynamics and dysfunction (Figures 2C–2E). Consistent with the result observed in the *in vitro* study (Figure 1G), upregulation of ROS (MitoSox flow cytometry) was observed in MITOL knockout cardiac mitochondria (Figure 2F). To further confirm the oxidative protein modifications, we examined protein carbonylation, which can be detected as a smear by western blotting, and proved subsequent accumulation of carbonylated proteins (Figures 2G and 2H). We next examined the effects of MITOL deletion on mitochondrial respiration. Both respiratory control ratio and state III respiration significantly decreased in MITOL-cKO mitochondria, indicating decreased respiration rate in MITOL-deficient cardiomyocytes (Figures 2I and S2F). Cytochrome c oxidase/succinate dehydrogenase double-staining indicated reduced respiratory chain activity in MITOL-cKO mice (Figure S2G). However, activities and protein components of each respiratory chain complex were comparable between control and MITOL-cKO mitochondria (Figures S2H and S2I), suggesting an impaired formation of respiratory chain supercomplex (Milenkovic et al., 2017). To confirm this, a Blue Native-PAGE assay was performed to detect respiratory chain supercomplex formation in cardiac mitochondria and revealed impaired supercomplex formation in MITOL-cKO mice (Figures S2J and S2K). A luciferase assay showed that ATP content was reduced in the hearts of MITOL-cKO mice (Figure 2J). Taken together, these results suggest that mitochondrial fragmentation by Drp1 accumulation because of the OMMAD dysfunction partly impaired mitochondrial functions, such as upregulation of oxidative stress in cardiomyocytes in MITOL-cKO mice.

### MITOL is indispensable for the maintenance of cardiac function

**Figure 3:**
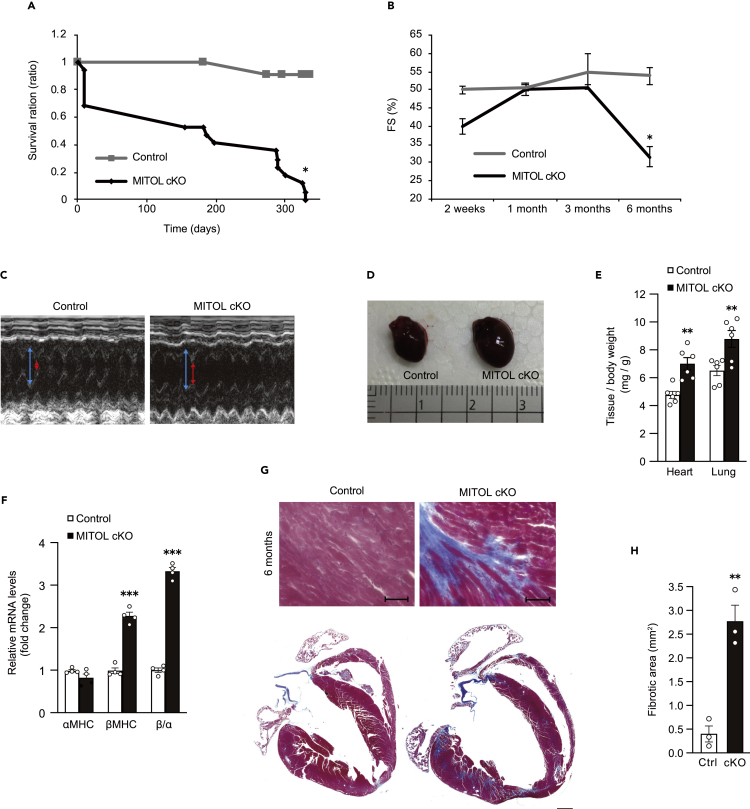
Cardiac ablation of MITOL caused heart failure with severe fibrosis (A) Kaplan-Meier survival curve of wild-type (WT: gray line) and tamoxifen-treated Mitol^f/f^;αMHC-Cre^Mer^ (MITOL cKO: black line) mice. Control = 25, MITOL cKO = 45 mice per group. ∗p < 0.05, Student’s t-test. (B) Cardiac dysfunction in MITOL-cKO mice. Echocardiographic analysis of left ventricular dimensions and cardiac function in mice. FS, fractional shortening of left ventricular diameter, calculated as [(LVIDd – LVIDs)/LVIDd] × 100. LVIDs; systolic left ventricular internal diameters. LVIDd; diastolic left ventricular internal diameters. Mean ±SEM (n = 3). ∗p < 0.05, Student’s t-test. (C) Representative M-mode echocardiograms from control and MITOL-cKO mice six months after tamoxifen treatment. Red arrow, LVIDs. Blue arrow, LVIDd. (D and E) Cardiac hypertrophy in MITOL-cKO mice. Hearts from control and cKO mice were isolated and photographed six months after tamoxifen-treatment. Scale represents 1 mm (D). Heart-to-body and Lung-to-body weight ratio in control and cKO mice six months after tamoxifen treatment. Mean ±SEM (n = 6). Analysis was performed with two-way ANOVA followed by Bonferroni *post hoc* analysis. ∗∗p < 0.01. (E). (F) Elevated levels of the hypertrophic markers in MITOL-cKO mice. The gene expression of αMHC and βMHC was analyzed by qRT-PCR. Bar graphs show the relative mRNA levels of αMHC and βMHC, and the ratio of βMHC to αMHC in MITOL-cKO mice compared with control mice. Mean ± SEM (n = 4). ∗∗∗p < 0.001., Student’s t-test. (G and H) Myocardial fibrosis in MITOL-cKO mice. Representative views of cross-sections of control and MITOL-cKO mice three and six months after tamoxifen-treatment, stained with Masson’s trichrome to detect fibrosis (G). Bars, 100 μm. The fibrotic areas were measured (H). Mean ±SEM (n = 3). ∗∗p < 0.01. Representative low-magnification views of cross-sections at the midventricle from cKO mice three and six months after tamoxifen-treatment (bottom).

We next assessed the cardiac function of MITOL-cKO mice. Initially, we analyzed survival rates of MITOL-cKO mice after tamoxifen treatment. Approximately 30% of mice died within two weeks following tamoxifen treatment, and almost the remaining mice died within one year (Figure 3A). We next analyzed systolic function by monitoring fractional shortening (FS) of the left ventricle with echocardiography. Six months post-tamoxifen treatment, FS of MITOL-cKO mice was dramatically lower compared with that of control mice (Figures 3B, 3C, and S3A). Accordingly, MITOL-cKO mice showed typical features of heart failure, including cardiac hypertrophy (enlarged myocardium, a high heart weight (HW)/body weight (BW) ratio), and changes in gene expression profiles (αMHC to βMHC) (Figures 3D–3F).

It has been reported that heart failure occurs as the result of cardiomyocytes apoptosis (van Empel et al., 2005). To examine this possibility, we assessed cardiac toxicity by Masson’s trichrome staining, TUNEL assays, and the immunodetection of cleaved caspase-3. Masson’s trichrome staining revealed massive fibrotic areas in MITOL-cKO myocardium six months post-tamoxifen treatment, indicating that significant fibrosis occurred (Figures 3G and 3H). Consistently, the hearts of MITOL-cKO mice showed increased TUNEL-positive cells and cleaved caspase-3 (Figures S3B–S3F). These results indicate that heart failure, accompanied by cardiomyocyte cell death, occurred in the hearts of MITOL-cKO mice.

### MITOL deletion-induced accumulation of senescent cardiomyocytes

**Figure 4:**
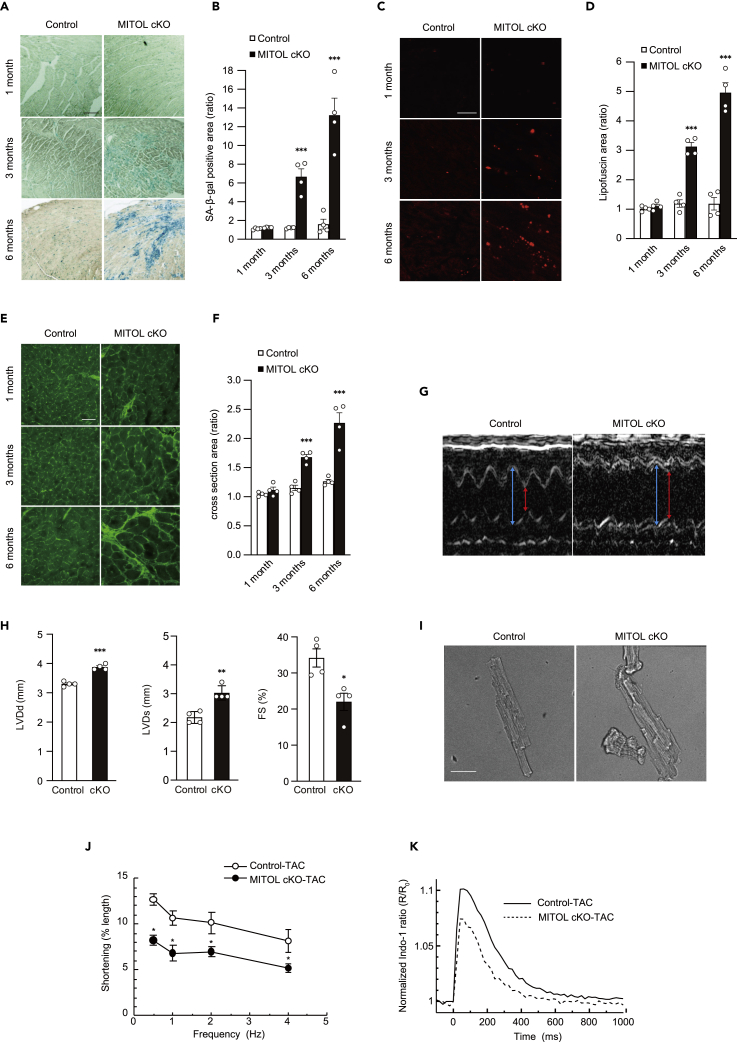
Cardiac ablation of MITOL caused heart aging and vulnerability to TAC treatment (A and B) Senescent cardiomyocytes were accumulated in MITOL-cKO mice. Senescent cells were detected by SA-β-Gal staining in the heart for the indicated periods after MITOL deletion by tamoxifen (A). Bar graph shows the percentage of SA-β-Gal positive cells in the heart sections (B). Mean ±SEM (n = 4). ∗∗∗p < 0.001. Bar, 200 μm. (C and D) Accumulation of lipofuscin and cardiomyocyte hypertrophy in MITOL-cKO mice. Frozen heart tissue sections were analyzed for lipofuscin by fluorescence microscopy. Lipofuscin was detected as autofluorescence (C). The relative content of lipofuscin (area of lipofuscin granules/field of the myocardium) was analyzed with Image J (D). Mean ±SEM (n = 4). ∗∗∗p < 0.001. Bar, 30 μm. (E and F) Frozen heart tissue sections were also stained with FITC-conjugated wheat germ agglutinin (WGA) to detect cardiomyocyte borders (E). The areas of cardiomyocytes were measured from 100 cells. Mean ±SEM (n = 4). ∗∗∗p < 0.001. Bar, 50 μm. Bar graphs show relative size of cells compared with control one month after tamoxifen-treatment (F). (G) Representative M-mode echocardiograms from control-TAC and MITOL-cKO-TAC mice. Mice for three months after tamoxifen injection were subjected to TAC surgery for two weeks. Red arrow, LVIDs. Blue arrow, LVIDd. (H) Impaired systolic function without cardiac hypertrophy in MITOL-cKO-TAC mice. Echocardiographic analysis of left ventricular dimensions and cardiac function in control-TAC and MITOL-cKO-TAC mice. The graph shows the percentage of FS. Mean ±SEM (n = 4). ∗p < 0.05, ∗∗p < 0.01, ∗∗∗p < 0.001, Student’s t-test. (I and J) Representative images of control-TAC and MITOL-cKO-TAC cardiomyocytes (I). Bar, 50 μm. Frequency-dependent shortening of cardiomyocytes isolated from control-TAC and MITOL-cKO-TAC mice (J). Mean ±SEM (n = 4). ∗p < 0.05. (K) Averaged traces of Ca^2+^ transients evoked at electric stimulation at 1 Hz. Indo-1 fluorescence ratio (R) was normalized to the averaged ratio before electric stimulation (R_0_).

Massive fibrosis is often associated with cardiac senescence, as well as abnormity of mitochondrial-dynamics-accelerated cardiomyocyte senescence, prematurely inducing the cardiomyopathy of aging (Chaudhary et al., 2011). In addition, both the accumulation of ROS and enhanced oxidative stress in MITOL-cKO cardiac mitochondria (Figures 2F–2H) prompted us to consider whether cellular senescence is accelerated in MITOL-cKO cardiomyocytes *in vivo*, as observed in the *in vitro* study (Figures 1H, 1I, S1E, and S1F). As shown in Figure 4A–4F, MITOL-cKO cardiomyocytes developed characteristics of senescence, including accumulation of β-gal positive cells (Figures 4A and 4B), deposition of lipofuscin (Figures 4C and 4D), and an increase in myocardium cross-sectional area (Figures 4E and 4F) three to six months post-tamoxifen treatment. Co-staining of lipofuscin and Troponin T confirmed that lipofuscin accumulated in the MITOL-cKO cardiomyocytes (Figure S4A). Together, these results suggest cardiomyocyte senescence is accelerated in the hearts of MITOL-cKO mice, and ROS may contribute to this process.

Accelerated senescence, with massive cardiomyocyte cell death, in MITOL-cKO mice led us to consider whether MITOL-cKO cardiomyocytes are vulnerable to cardiac stress. Transverse aortic constriction (TAC) is a well-established model of pressure overload-induced heart failure in mice (Rockman et al., 1991), and cellular senescence is associated with heart failure (Childs et al., 2018; Shimizu and Minamino, 2019). To test the vulnerability of MITOL-cKO hearts, we performed transverse aortic constriction (TAC) surgery on control and MITOL-cKO mice after three months of tamoxifen administration, when the MITOL deficient mice still maintain normal cardiac function. We then examined cardiac function after two weeks of TAC surgery. Representative M-mode echocardiograms on control-TAC and MITOL-cKO-TAC mice, two weeks after TAC surgery, are shown in Figure 4G. MITOL-cKO mice displayed significantly more serious cardiac damage to TAC surgery than control mice, as observed by impairment of systolic function (Figure S4B, Figure 4H). In addition, MITOL-cKO-TAC mice showed typical features of cardiac hypertrophy, including a trend toward a higher HW/BW ratio (Figure S4C).

Because mechanical impairment at the level of individual cardiomyocytes is correlated with global cardiac dysfunction, we analyzed cell shortening and Ca^2+^ handling of single myocytes isolated from MITOL-cKO-TAC and control-TAC mice. Cardiomyocytes isolated from MITOL-cKO-TAC mice displayed hypertrophy with a ragged shape, suggesting accelerated senescence (Figure 4I). Under field stimulation at 0.5–4 Hz, cardiomyocytes of MITOL-cKO-TAC shortened significantly less than in control-TAC at all frequencies (0.5, 1, 2, 4 Hz) (Figure 4J). The peak amplitude of electrically-evoked Ca^2+^ transient was significantly smaller in MITOL-cKO-TAC versus control-TAC myocytes (Figures 4K and S4D), although sarcoplasmic reticulum Ca^2+^ content was not statistically different (Figures S4E and S4F). These results indicate that the reduction in contractility at the individual cell level, because of the decrease in peak amplitude of Ca^2+^ transient is a cause of global cardiac dysfunction in MITOL-cKO-TAC mice. Representative photographs show enhanced myocardial fibrosis in MITOL-cKO-TAC mice (Figure S4G). Together, MITOL deletion caused cardiac senescence and high vulnerability to cardiac stress.

### Drp1 accumulation is partly responsible for cardiac damages in MITOL cKO mice

**Figure 5:**
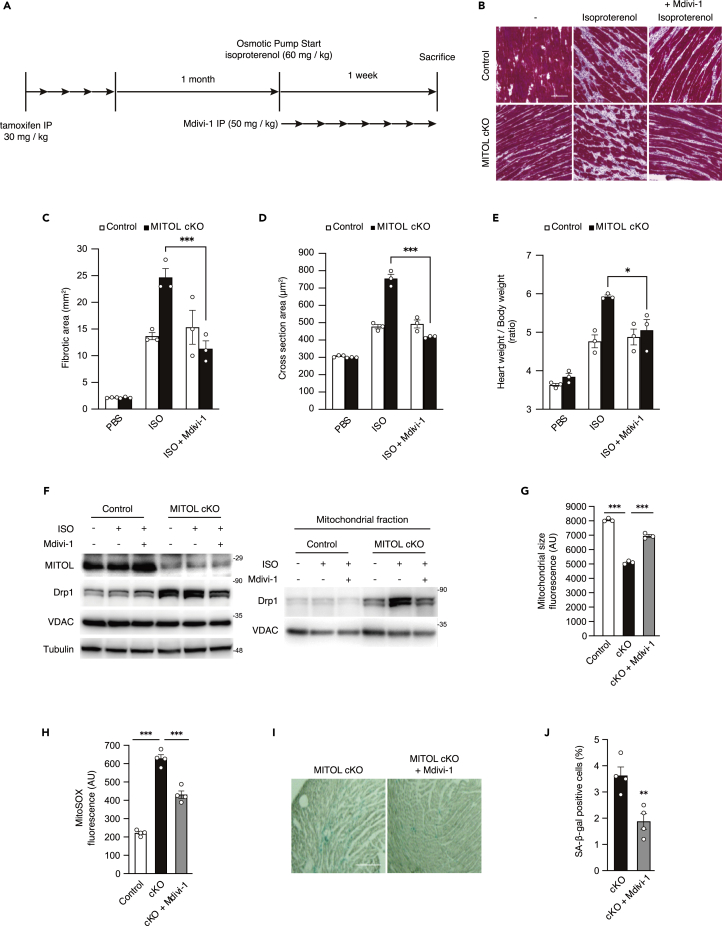
Mdivi-1 restored the vulnerability of MITOL-KO hearts (A) Study design for determining cardiac stress resistance in MITOL-cKO mice. Mice were intraperitoneally (i.p.) administered five consecutive days of treatment with tamoxifen (30 mg/kg) once daily. One month after administration of tamoxifen, mice were administered with isoproterenol (60 mg/kg) via subcutaneous infusion from implanted osmotic pumps. Seven days after infusion of isoproterenol, mice were then assessed for cardiac function. For Drp1 inhibition, mice were i.p. administered seven consecutive days of treatment with Mdivi-1 (50 mg/kg) once daily. (B and C) Myocardial fibrosis in MITOL-cKO mice under isoproterenol (ISO) stress was attenuated by Mdivi-1. Representative photographs show Masson’s trichrome staining for collagen (B). Bar, 200 μm. The fibrotic areas were measured (C) Mean ± SEM (n = 3). Analysis was performed with two-way ANOVA followed by Bonferroni *post hoc* analysis. ∗∗∗p < 0.001.. (D and E) Cardiomyocyte hypertrophy in MITOL-cKO mice under isoproterenol stress was attenuated by Mdivi-1. Frozen heart tissue sections were also stained with FITC-conjugated wheat germ agglutinin (WGA) to detect cardiomyocyte borders. The areas of cardiomyocytes were measured from 100 cells. Mean ±SEM (n = 3) (D). Heart-to-body and weight ratio in control and MITOL-cKO mice. Mean ±SEM (n = 3) (E). ∗p < 0.05, ∗∗∗p < 0.001. (F) Mdivi-1 attenuates Drp1 accumulation in MITOL-cKO mouse cardiomyocytes. Lysates from total heart cells of control mice, and MITOL-cKO mice treated with or without Mdivi-1 under isoproterenol (ISO) stress were subjected to immunoblotting with respective antibodies. Cardiac mitochondrial fractions of MITOL-cKO mice treated with or without Mdivi-1 (described in A) were subjected to immunoblotting with respective antibodies. (G) Decreased size of mitochondria in MITOL-cKO was recovered by Mdivi-1 administration. Cardiac mitochondrial areas of control mice, and MITOL-cKO mice treated with or without Mdivi-1 (described in A) were measured. Mitochondrial fractions isolated from cardiomyocytes were stained with MitoTracker, followed by flow cytometric analysis. Mean ±SEM (n = 3). Analysis was performed with one-way ANOVA followed by Bonferroni *post hoc* analysis. ∗∗∗p < 0.001. (H) Attenuation of mitochondrial ROS in MITOL-cKO by Mdivi-1 administration. Cardiac mitochondrial fractions of control mice, and MITOL-cKO mice treated with or without Mdivi-1 (described in A) were stained with MitoSOX and mitochondrial-derived superoxide generation was measured by flow cytometric analysis. Bar graphs show the mean fluorescence intensity of MitoSOX. Mean ±SEM (n = 4). Analysis was performed with one-way ANOVA followed by Bonferroni *post hoc* analysis. ∗∗∗p < 0.001.. (I and J) Increased senescent cardiomyocytes in MITOL-cKO were recovered by Mdivi-1 administration. Senescent cells were detected by SA-β-Gal staining in the heart for the indicated periods after MITOL deletion by tamoxifen (I). Bar, 200 μm. Bar graph shows the percentage of SA-β-Gal positive cells in the heart sections (J). Mean ±SEM (n = 4). ∗∗p < 0.01.

To estimate the contribution of Drp1 accumulation on cardiac dysfunction and accelerated senescence by MITOL deletion, we examined whether Mdivi-1 could rescue these phenotypes in MITOL-cKO mice. Because it takes a long time to see the effects of Mdivi-1 without stress, mice were subjected to cardiac load by infusion of isoproterenol. Isoproterenol, a β-adrenergic agonist, causes heart disease in experimental mice with mitochondrial dysfunction, which triggers the intrinsic pathway of apoptosis (Acin-Perez et al., 2018; Mukherjee et al., 2012). The study design is schematically shown in Figure 5A. By consecutive infusion of isoproterenol for seven days using a subcutaneous mini-osmotic pump, hearts of control mice developed interstitial fibrosis, a typical feature of heart failure (Figure 5B, above center, 5C). As expected, isoproterenol infusion leads to increased interstitial fibrosis and cardiac hypertrophy in MITOL-cKO mice (Figures 5B–5E). Concomitantly, aberrant accumulation of Drp1 to mitochondria, the reduction of mitochondrial size, and ROS production were observed in MITOL-cKO mice (Figures 5F–5H).

We examined the involvement of Drp1 in the vulnerability of MITOL-cKO hearts. To explore this, coadministration of isoproterenol and Mdivi-1 to MITOL cKO mice was performed. Administration of Mdivi-1 partially rescued accumulation of Drp1 and abnormal mitochondrial size, indicating an inhibitory effect of Mdivi-1 on mitochondrial translocation of Drp1 (Figures 5F and 5G). We, therefore, judged Mdivi-1 could work *in vivo* as a Drp1 inhibitor. Administration of Mdivi-1 partially rescued ROS production (Figure 5H), suggesting attenuation of Drp1 toxicity on mitochondria. Consistently, Mdivi-1 also blocked interstitial fibrosis, cardiomyocyte hypertrophy, and increased heart weight in MITOL-deficient hearts (Figures 5C–5E). Furthermore, senescent cardiomyocytes decreased significantly by the Mdivi-1 treatment (Figures 5I and 5J).

In addition to Drp1 inhibition, Mdivi-1 has been reported to have a side effect on the mitochondrial respiratory complex 1 (Bordt et al., 2017). Therefore, we examined whether adeno-associated virus vector (AAV)-mediated delivery of a dominant-negative Drp1 (K38A-Drp1) into cardiomyocytes decreased cell death by MITOL deletion (Figures S5A and S5B). Myocardial fibrosis in MITOL-cKO mice was attenuated by AAV-K38A-Drp1 (Figure S5C). AAV-K38A-Drp1 delivery also blocked cardiomyocyte hypertrophy and the increase of heart weights in MITOL-deficient hearts (Figures S5D and S5E). Furthermore, the senescent marker lipofuscin was significantly reduced by AAV-K38A-Drp1 (Figure S5F).

Taken together, these data suggest that the vulnerability and senescence of cardiomyocytes is the cause of heart failure in MITOL-cKO mice, and Drp1 toxicity because of the OMMAD dysfunction may be involved, at least partially, in this process.

### Ischemic stress induces MITOL downregulation and enhances the vulnerability of cardiomyocytes

**Figure 6:**
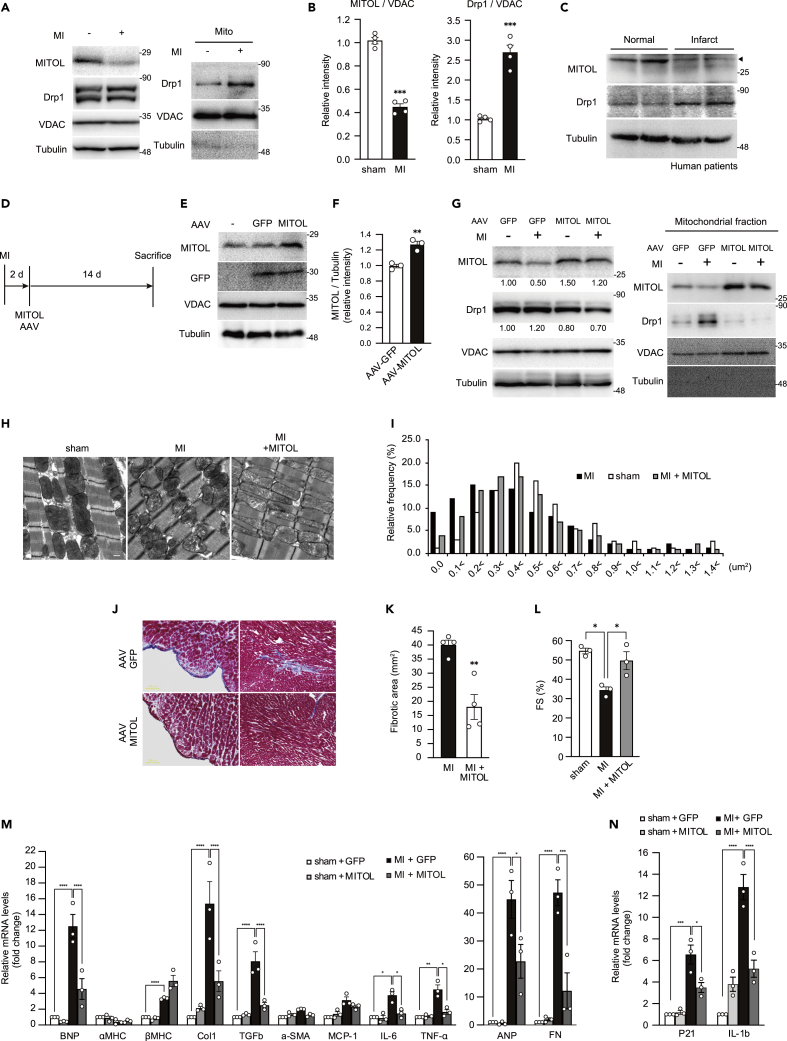
Downregulation of MITOL in infarct cardiomyocytes and transduction of AAV-MITOL ameliorates cardiac fibrosis under myocardial infarction (A and B) Downregulation of MITOL in infarct rat cardiomyocytes. MI was induced by ligation of the left coronary artery in rats. After two weeks of MI, IB assay of MITOL and Drp1 was performed on lysates of whole and mitochondrial fractions isolated from rat hearts. Anti-VDAC and anti-tubulin antibodies were used as a mitochondrial marker and a cytosolic marker, respectively. Data are standardized to VDAC levels and are expressed relative to sham operated rats. Mean ±SEM (n = 4). ∗∗∗p < 0.001, Student’s *t*-test. (C) MITOL decreased in infarct cardiomyocytes of humans. Lysates from total heart cells of normal and infarct left ventricle were immunoblotted with anti-MITOL, anti-Drp1 and anti-tubulin antibodies. Age: 67–70. (D) Study design for the transduction of MITOL in MI-induced cardiomyocytes. Two days after the induction of MI, rats were introduced with AAV vectors (GFP: AAV-GFP and GFP-P2A-MITOL: AAV-MITOL) packaged in AAV-9 capsids via intrathoracic injection. Two weeks after virus injection, hearts were dissected and the expression of GFP and MITOL was assessed by immunoblotting. (E and F) MITOL was upregulated in cardiomyocytes of rats transduced with AAV-MITOL. IB assay of MITOL, GFP and tubulin was performed on lysates of total rat heart cells (E). The relative protein levels of MITOL and GFP were quantified by densitometry (F). Data are standardized to tubulin levels and are expressed relative to AAV-GFP injected rats. Mean ± SEM (n = 3). ∗∗p < 0.01. (G) MI-induced MITOL downregulation was rescued by AAV-MITOL. After MI or sham operation, rats were injected with indicated viruses (as described in D). Two weeks after injection of viruses, IB assay of MITOL, Drp1 and VDAC were performed on lysates of whole and mitochondrial fractions of rat cardiomyocytes. Data are standardized to VDAC levels and are expressed relative to AAV-GFP injected rats. (H and I) Mitochondria damage induced by MI was rescued by AAV-MITOL. Representative electron microscopic images of mitochondria from the hearts of sham-operated, MI treated, and MI +AAV-MITOL-treated mice. 27,600-fold magnification (H). Bar, 500 nm. The distribution of mitochondrial size is shown (I). (J and K) Myocardial fibrosis was attenuated in MI-treated mice transduced with AAV-MITOL. Representative photographs show Masson’s trichrome staining for collagen(J). Mean ±SEM (n = 4) (K). ∗∗p < 0.01. (L) Cardiac malfunction was restored in MI-treated mice transduced with AAV-MITOL. Echocardiographic analysis of left ventricular dimensions and cardiac function in mice. FS, fractional shortening of left ventricular diameter. Mean ±SEM (n = 3). Analysis was performed with one-way ANOVA followed by Bonferroni *post hoc* analysis. ∗p < 0.05. (M) Gene expression of markers of cardiac function and hypertrophy. Relative mRNA abundance of Tumor Necrosis Factor α (TNF-α), atrial natriuretic peptide (ANP), brain natriuretic peptide (BNP; B), α-myosin heavy chain (α-MHC), β-MHC, collagen-1 (Col-1), MHC-β, α-smooth muscle actin (α-SMA), transforming growth factor β (TGF-β), and fibronectin (FN) in the myocardium of control mice and MITOL cKO mice treated with AAV-GFP or with AAV-MITOL tested by real-time qPCR. Data are presented as mean ± SEM. ∗p < 0.05 vs. control. Analysis was performed with one-way ANOVA followed by Bonferroni *post hoc* analysis. ∗p < 0.05, ∗∗p < 0.01, ∗∗∗p < 0.001, ∗∗∗∗p < 0.0001. (N) Gene expression of markers of cardiac aging. Relative mRNA abundance of P21 and Interleukin-1 (IL-1b) in the myocardium of control mice and MITOL cKO mice treated with AAV-GFP or with AAV-MITOL tested by real-time qPCR. Data are presented as mean ± SEM. ∗p < 0.05 vs. control. Analysis was performed with one-way ANOVA followed by Bonferroni *post hoc* analysis. ∗p < 0.05, ∗∗∗p < 0.001, ∗∗∗∗p < 0.0001.

Mitochondrial dysfunction plays a key role in the pathogenesis of ischemic heart disease (Walters et al., 2012). Therefore, we examined the pathophysiological significance of the OMMAD by MITOL in ischemic heart. We used a MI model to confirm MITOL downregulation and Drp1 accumulation under an ischemic condition *in vivo*. Downregulation of MITOL and accumulation of Drp1 in mitochondria was observed in the nonfibrotic area of infarcted rat hearts (Figures 6A and 6B). Furthermore, MITOL downregulation was observed in infarcted hearts of humans and mice (Figures 6C and S6A). Immunostaining revealed that the downregulation was occurred in cardiomyocytes of the peri-infarcted region (Figure S6B). These results indicate that MITOL is downregulated under ischemic conditions pathologically. To investigate whether MITOL downregulation elicited by MI contributes to cardiac dysfunction, we attempted to overexpress MITOL in cardiomyocytes by intrathoracic injection of adeno-associated virus. The study design is schematically shown in Figure 6D.

To enhance cardiac tropism, we inserted the cardiac-specific troponin T (cTnT) promoter upstream of AAV-9. After the intrathoracic injection of the virus to rats, we could confirm the significant expression of GFP and MITOL in cardiomyocytes (Figures 6E and 6F). We next injected the virus into infarcted rats and monitored MITOL expression. MITOL expression was downregulated in infarcted rats injected with the control virus, whereas the downregulation of MITOL was circumvented in rats injected with AAV-MITOL (Figure 6G). Electron microscopy revealed mitochondrial fragmentation in infarcted rat cardiomyocytes (Figure 6H), partially recovered after AAV-MITOL treatment (Figures 6H and 6I). Furthermore, AAV-MITOL treatment reduced cardiac hypertrophy and interstitial cardiac fibrosis after MI (Figures 6J, 6K, and S6C). Accordingly, the impairment of systolic function after MI was partially recovered by AAV-MITOL treatment (Figure 6L). In addition, marker gene expressions of cardiac dysfunction, hypertrophy, and senescence were restored in the AAV-MITOL-treated hearts (Figures 6M and 6N). These results suggest that the downregulation of MITOL is one of the causes of cardiac damage in MI; the fact that ectopic expression of MITOL rescued the cardiac damage indicates that the OMMAD activation by MITOL can be a therapeutic target for MI.

## Discussion

In this study, we showed that the regulation of mitochondrial morphology by MITOL’s OMMAD activity is physiologically critical for maintaining cardiac functions because of its antiaging effect *in vivo*. We also demonstrated that MI induces the downregulation of MITOL, and the overexpression of MITOL restored ischemic stress-triggered mitochondrial fragmentation and heart failure. These results indicate that MITOL is downregulated to suppress energy production in response to ischemic stresses, but the excessive and prolonged downregulation of MITOL exerts cardiotoxicity leading to heart failure. We found that MITOL regulates mitochondrial morphology in cardiac cells *in vivo*, mainly depending on Drp1. In particular, Drp1-dependent excess mitochondrial fission by MITOL deletion, at least in part, contributes to cardiac damage leading to heart aging and cardiac cell death in pathological conditions like MI. Our observations may provide a better understanding of heart diseases' molecular pathology, including chronic heart failure and ischemic heart diseases.

We previously demonstrated that MITOL degrades Drp1 as a substrate, thereby preventing mitochondrial fragmentation (Yonashiro et al., 2006). On the other hand, other groups have reported that MITOL is required for Drp1 activation (Karbowski et al., 2007; Park et al., 2010). It was still controversial whether MITOL promotes Drp1 degradation or activates Drp1. This study demonstrated that Drp1 is a physiological substrate of MITOL. Among other mitochondrial division factors including Fis1, MFF, MID49, and MID51 (Fang et al., 2012; Gandre-Babbe and van der Bliek, 2008; Palmer et al., 2011; Xu et al., 2016), Fis1 and MID49 were reported to be substrates for MITOL (Xu et al., 2016; Yonashiro et al., 2006); it remains unestablished how MITOL controls mitochondrial morphology *in vivo*. We found that Drp1 serves as a central molecule in the MITOL-dependent mitochondrial morphology in the heart, rather than other substrates for MITOL. Considering that MITOL also interacts with Drp1 receptors, MITOL may specifically recognize and control the receptor-binding form of Drp1. Indeed, MITOL deletion increased the protein level of the Drp1 receptor MID49, in addition to Drp1, *in vivo*. Although it remains unknown how Drp1 selects its receptors, these observations led us to hypothesize that MITOL selectively degrades Mid49-bound Drp1.

Although mitochondrial dynamics in normal cardiomyocytes appear relatively stable compared to other types of cells (Dorn, 2015), perinatal and adult cardiomyocyte-specific gene deletion of mitochondrial dynamic proteins displayed requirements for these mitochondrial factors to maintain cardiac functions (Archer, 2013; Chen et al., 2011; Song et al., 2015; Varanita et al., 2015; Wai et al., 2015), indicating a critical role of mitochondrial dynamics in cardiomyocytes. It has also been reported that mitochondrial fragmentation is associated with pathological conditions and mitochondrial dysfunctions. For example, fragmented mitochondria in cardiomyocytes also have been observed in cardiomyopathy patients (Schaper et al., 1991), and pharmacological inhibition of Drp1 by Mdivi-1 attenuates heart failure in mice stressed by ischemia/reperfusion (Ong et al., 2010; Sharp, 2015), pressure overload (Chang et al., 2013; Givvimani et al., 2012), and doxorubicin (Gharanei et al., 2013), suggesting that abnormal mitochondrial fission caused by Drp1 in cardiomyocytes leads to the progression of heart failure, and that inhibition of Drp1 activity is an attractive target for heart failure. Our study further supports these reports and suggests that Drp1 regulation by MITOL is a key step in maintaining physiological cardiac functions and in pathological conditions. On the other hand, it has been reported that mitochondrial fragmentation because of artificial overexpression of Drp1 does not deteriorate cardiac function (Song et al., 2017). This report suggests that the accumulation of Drp1 is not highly toxic to cardiomyocytes. It is possible that the adverse effects because of MITOL deficiency are the cause of heart failure in MITOL cKO, as MITOL has several substrates other than Drp1. In this study, we only found Drp1 and Mid49 are the two proteins to change with MITOL deficiency (Figure S2D). Overall, we demonstrated that Drp1 is, at least in part, the cause of heart failure in MITOL cKO mice, but other effectors are still unknown. MITOL also regulates multiple cascades, including apoptosis, mitophagy, endoplasmic reticulum stress, and immune signaling (Arai et al., 2020; Chen et al., 2017; Shiiba et al., 2021; Takeda et al., 2019; Yoo et al., 2015). Given the importance of MITOL/MARCH5, cascades other than Drp1 may be involved in the MITOL cKO heart failure.

This is the first report to demonstrate the MITOL-Drp1-Senescence pathway *in vivo*. Antiapoptotic roles of MITOL against hydrogen peroxide have been previously suggested in HL-1 and neonatal rat cardiomyocytes *in vitro* (Wang et al., 2016). Although ROS is increased during MI, MITOL expression was not increased but decreased as opposed to the previous paper. This might be because of the differences between physiological ischemic stimuli and hydrogen peroxide. Another possibility is the differences between cultured cardiomyocytes and cardiac myocytes. As mitochondria in cultured cardiomyocytes from those in the myocardium are distinct (Song et al., 2015), our results provide the tissue-based results of MITOL roles for investigating MITOL-Drp1-mitochondrial regulation.

Chronic heart failure after MI is associated with excess mortality (Ezekowitz et al., 2009; Kostis et al., 2010; Velagaleti et al., 2008). It has been reported that the cytoskeletal regulator filamin increases the mitochondrial fission activity of Drp1, which potentially contributes to the cardiotoxicity of MI (Nishimura et al., 2018). In this study, we demonstrated for the first time an upstream mechanism of Drp1 degradation by MITOL in MI. Our data indicate that the downregulation of MITOL partly triggers Drp1 cytotoxicity in MI. Consistently, the cardiotoxicity of MI was counteracted by MITOL expression by adenovirus-mediated gene transfer (Figures 6J–6N). Therefore, induction of MITOL expression may be a good therapeutic target to attenuate cardiac dysfunctions in MI. However, how MITOL is downregulated in response to ischemic stress is still not fully understood.

MITOL-cKO mice had slightly reduced cardiac function in the early stage (Figure 3B). Although these cardiac dysfunctions recover over time, the cardiotoxicity of early MITOL deficiency is not well understood. We did not observe any cardiac fibrosis or cell death, at least in the early MITOL-cKO (data not shown). There could be some redundancy in recovery of cardiac function.

Although Drp1 and Mfn1/2 are candidates for treating heart failure by modulating mitochondrial morphology, these molecules are critical and fundamental regulators for maintaining mitochondrial morphology and functions. Therefore, overexpression or downregulation of these molecules may lose even beneficial responses or exert side effects such as mitochondrial damages, making them suboptimal as therapies. On the other hand, MITOL indirectly regulates mitochondrial morphology through the OMMAD activity as a guardian. Therefore, even when MITOL is overexpressed, it would degrade only molecules that damage the mitochondria. MITOL overexpression simply increases the OMMAD activity and improves OMM clearance. Thus, compensating for the downregulated MITOL in MI can be an attractive therapeutic strategy. In the near future, we expect to develop innovative drugs that can suppress MITOL downregulation.

In conclusion, our findings identify MITOL as a good therapeutic target for MI or aging-related diseases, and that it may be possible to find innovative drugs based on the MITOL-Drp1 signaling pathway on the OMM.

### Limitations of the study

In this study, we have generated and analyzed myocardial-specific MITOL-deficient mice. We found that the mutant mice display severe heart failure and die within 6 months after MITOL deletion. We demonstrated that MITOL is essential for cardiac function, at least in part, through the Drp1 regulation. However, it is considered that there are multiple possible causes, because MITOL ubiquitinates not only Drp1 but also many other substrates that play important roles in mitochondrial function. We focused on Drp1 accumulation in the MITOL-deleted heart and used Mdivi-1, an inhibitor of Drp1, to suggest that Drp1 accumulation is one of the main causes of cardiac dysfunction because of MITOL deficiency. Unfortunately, we could not prove whether Mdivi-1 really acts specifically against Drp1 *in vivo*. A limitation of this study is that the specificity of Mdivi-1 for Drp1 remains unresolved. This problem would be solved if a mutant mouse could be generated by knocking into the Drp1 genome, a Drp1 mutant in which the lysine site of Drp1, which is ubiquitinated by MITOL, is substituted to arginine residue. However, generating such mutant mice is technically very difficult and requires an enormous amount of time. Realistically, we hope that several Drp1 specific inhibitors will be developed.

## STAR★Methods

### Key resources table

**Table:** 

REAGENT or RESOURCE	SOURCE	IDENTIFIER
**Antibodies**		

Anti-MITOL rabbit polyclonal antibody	In house	N/A
Anti-FLAG-M2	Sigma	F-1804; RRID: AB_262044
Anti-αActinin	Sigma	A-7732; RRID: AB_2221571
Anti α-tubulin	Sigma	T-9026; RRID: AB_477593
Anti-HA antibody	MBL	M132-3; RRID: AB_10207271
Antibodies against Drp1	BD Biosciences	#611112; RRID: AB_398423
Antibodies against Tom20	BD Biosciences	#612278; RRID: AB_399595
Anti-GFP antibody	MBL	M048-3; RRID: AB_591823
Anti-NDUFA9 antibody	Invitrogen	#459100; RRID: AB_2532223
Anti-COX4	Invitrogen	PA5-29992; RRID: AB_2547466
Anti-Troponin T	Abcam	ab8295; RRID: AB_306445
Anti-Drp1	Abcam	ab56788; RRID: AB_941306
Anti-ATP5A	Abcam	ab14748; RRID: AB_301447
Anti-UQCRC2	Abcam	ab14745; RRID: AB_2213640
Anti-VDAC	Cell Signaling	#4866; RRID: AB_2272627
Anti-Caspase-3	Cell Signaling	#9662; RRID: AB_331439
Anti-cleaved Caspase3	Cell Signaling	#9661; RRID: AB_2341188
Anti-SDHA	Cell Signaling	11998S; RRID: AB_2750900
Mitochondrial Marker Antibody Sampler Kit	Cell Signaling	#8674; RRID: AB_11217817
Mitochondrial Dynamics Antibody Sampler Kit II	Cell Signaling	#74792
Anti-GAPDH	Acris Antibodies	ACR001-PT; RRID: AB_1616730

**Biological samples**		

Human adult normal heart tissue lysate	BioChain	P1234138
Human adult normal heart tissue lysate	BioChain	P1234122
Human adult infarct tissue lysate	BioChain	P1236138Hd-1
Human adult infarct tissue lysate	BioChain	P1236122Hd-1

**Chemicals, peptides, and recombinant proteins**		

4-OHT	Sigma	H7904
Tamoxifen	Cayman Chemical	#13258
Isoproterenol	nacalai tesque	#19703-04
Digitonin	WAKO	#044-02121

**Critical commercial assays**		

RNeasy kit	QIAGEN	#74134
QuantiTect Reverse Transcription Kit	QIAGEN	#205311
THUNDERBIRD SYBR qPCR Mix	TOYOBO	QPS-201
Masson’s trichrome staining	Sigma	HT15-1KT
Senescence β-Galactosidase Staining Kit	Cell Signaling	#9860S
Protein carbonyl kit	SHIMA Laboratories	#ROIK03
luciferase-based ATP determination kit	TOYO B NET	TA100
*In Situ* Cell Death Detection Kit	Roche	11684795910

**Experimental models: Cell lines**		

MITOL^flox/flox^ MEFs	Sugiura et al. (2013)	N/A

**Experimental models: Organisms/strains**		

Mouse: MITOL^flox/flox^	Sugiura et al. (2013)	N/A
Mouse: αMHC-MerCreMer	Sohal et al. (2001)	N/A
Rat: Slc:Wistar	SLC	N/A

**Oligonucleotides**		

All qPCR primer sequences listed in Table S1 in Method details.		

**Recombinant DNA**		

pAAV/cTnT-GFP-P2A-MITOL-WPRE	This paper	N/A
pAAV/cTnT-GFP-P2A-K38A Drp1-WPRE	This paper	N/A

**Software and algorithms**		

Fiji	ImageJ	https://imagej.net/software/fiji/downloads
Prism	GraphPad	https://www.graphpad.com/
Adobe Photoshop	Adobe	https://www.adobe.com/es/
Adobe Illustrator	Adobe	https://www.adobe.com/es/
ImageJ	ImageJ	https://imagej.nih.gov/ij/

### Resource availability

#### Lead contact

Further information and requests for resources and reagents should be directed to and will be fulfilled by the lead contact, Shigeru Yanagi (shigeru.yanagi@gakushuin.ac.jp).

#### Materials availability

This study did not generate new unique reagents.

### Experimental model and subject details

All animals were maintained under university guidelines for the care and use of animals. The experiments were performed after securing Tokyo University of Pharmacy and Life Sciences Animal Use Committee Protocol approval. Tamoxifen-induced Cre LoxP recombination was activated by intraperitoneal administration of tamoxifen (30 mg/kg) for 5 days. Cre-negative assorted littermates, also treated with tamoxifen, served as controls. Sham control rats and myocardial infarction model rats were purchased from Japan SLC. 8- to 12-week-old male MITOL KO mice were randomly assigned to receive i.p. injections of either isoproterenol (60 mg/kg, #19703-04, nacalai tesque) or saline for 7 days using mini-osmotic pumps (model: alzet-20; Durect Corporation, CA. USA). Mice received once daily i.p. injections (50 mg/kg) with mdivi-1 beginning on the day of the first isoproterenol treatment and continued until mice were collected 7 days.

### Method details

#### DNA constructs

MITOL expression vectors were constructed by PCR using appropriate primers and subcloned into the pCMV5 vector (Sugiura et al., 2013). Drp1-FLAG and Drp1-3xFLAG expression vector were obtained by subcloning mCh-Drp1 (purchased from Addgene #49152).

#### Cell culture and transfection

MEFs were maintained in Dulbecco’s modified Eagle’s medium (DMEM) supplemented with 10% fetal bovine serum (FBS) and penicillin/streptomycin. Cells were transfected with Lipofectamine 3000 or RNAiMax (#13778150, Invitrogen) according to the manufacturer’s instructions. The following siRNAs were used: siDrp1, FlexiTube siRNA GS74006 (QIAGEN). MITOL^flox/flox^ MEFs derived from MITOL^flox/flox^ mice (Sugiura et al., 2013). MITOL was completely knocked out in MITOL^flox/flox^ MEFs 2 days after treatment with 0.8 μM 4-hydoroxytamoxifen (4-OHT, H7904, Sigma). All animal experiments were approved by the Animal Ethical Committee of Tokyo University of Pharmacy and Life Sciences.

#### Antibodies and reagents

Anti-MITOL rabbit polyclonal antibody was produced by immunizing rabbits with a synthetic peptide, GCKQQQYLRQAHRKILNYPEQEEA, corresponding to amino acids 257–279 of MITOL (Yonashiro et al., 2006). Anti-FLAG-M2 (F-1804) and mouse monoclonal anti α-tubulin (T-9026) antibodies were purchased from Sigma. The mouse monoclonal anti-HA antibody (M132-3) was from MBL. The mouse monoclonal antibodies against Drp1 (#611112) and Tom20 (#612278) were from BD Biosciences. Mouse monoclonal anti-GFP antibody (M048-3) was from MBL. Rabbit polyclonal anti-Tom20 (FL-145) antibody was from Santa Cruz (sc-11415). Mouse monoclonal anti-NDUFA9 antibody was from Invitrogen (#4591000). Rabbit polyclonal anti-COX4 antibody was from Invitrogen (PA5-29992). The mouse monoclonal antibodies against Troponin T (ab8295), Drp1 (ab56788), ATP5A (ab14748) and UQCRC2 (ab14745) were from Abcam. The rabbit polyclonal antibodies against VDAC (#4866), Caspase-3 (#9662) and cleaved Caspase3 (#9661) were from Cell Signaling. Rabbit monoclonal anti-SDHA antibody was from Cell Signaling (11998S). Mitochondrial Marker Antibody Sampler Kit (#8674) and Mitochondrial Dynamics Antibody Sampler Kit II (#74792) were from Cell Signaling. Mouse monoclonal anti-GAPDH antibody was from Acris Antibodies (ACR001-PT). 4-OHT (H7904, Sigma) was dissolved in ethanol (EtOH) and used for cellular experiments at 0.4 μM. Ethanol was also treated with cells as control.

#### RNA isolation and qRT-PCR

Total RNA was isolated from mammalian cells using RNeasy kit (#74134, QIAGEN) and subjected to reverse transcription to cDNA using QuantiTect Reverse Transcription Kit (#205311, QIAGEN), following the manufacturer’s protocol. PCR was performed using a THUNDERBIRD SYBR qPCR Mix (QPS-201, TOYOBO). The PCR conditions were as follows: 95°C for 1 min followed by 40 cycles at 95°C for 15 s, 60°C for 30 s, and 72°C for 60 s.

The following primers were used: *aMHC:* forward, 5′- GGAAGAGTGAGCGGCGCATCAAGG -3′, reverse, 5′- CTGCTGGAGAGGTTATTCCTCG -3′; *bMHC:* forward, 5′- GCCAACACCAACCTGTCCAAGTTC-3′, reverse, 5′- TGCAAAGGCTCCAGGTCTGAGGGC-3′; *GAPDH:* forward, 5′- TGGTGAAGCAGGCATCTGAG-3′, reverse, 5′- CTCCTGCGACTTCAACAGCA-3′; *ANP:* forward, 5′- AGGATTGGAGCCCAGAGTGGACTAGG-3′, reverse, 5′- TGATAGATGAAGGCAGGAAGCCGC-3′; *BNP:* forward, 5′- ATGGATCTCCTGAAGGTGCTG-3′, reverse, 5′- GTGCTGCCTTGAGACCGAA-3′; *Col-1:* forward, 5′- TGTTCGTGGTTCTCAGGGTAG-3′, reverse, 5′- TTGTCGTAGCAGGGTTCTTTC-3′; *TGFb:* forward, 5′- GCAACAACGCAATCTATGAC-3′, reverse, 5′- CCTGTATTCCGTCTCCTT-3′; *a-SMA1:* forward, 5′- CTGGAGAAGAGCTACGAACTGC-3′, reverse, 5′- CTGATCCACATCTGCTGGAAGG-3′; *MCP1:* forward, 5′- TTAAAAACCTGGATCGGAACCAA-3′, reverse, 5′- GCATTAGCTTCAGATTTACGGGT-3′; *IL-6:* forward, 5′- TAGTCCTTCCTACCCCAATTTCC-3′, reverse, 5′- TTGGTCCTTAGCCACTCCTTC-3′; *TNF-a:* forward, 5′- TGATCCGCGACGTGGAA-3′, reverse, 5′- ACCGCCTGGAGTTCTGGAA-3′; *FN:* forward, 5′- TGTGACAACTGCCGTAGACC-3′, reverse, 5′- GACCAACTGTCACCATTGAGG-3′; *p21:* forward, 5′- ATGTCCAATCCTGGTGATGT-3′, reverse, 5′- TGCAGCAGGGCAGAGGAAGT-3′; *p16:* forward, 5′- CAGATTCGAACTGCGAGGA-3′, reverse, 5′- CAGCGGAACACAAAGAGCA-3′.

#### Production of AAV9 vectors

Recombinant AAV9 vectors were generated by the co-transfection of HEK293T cells with three plasmids, including pAAV/cTnT-GFP-P2A-WPRE or pAAV/cTnT-GFP-P2A-MITOL-WPRE, pHelper (Stratagene, La Jolla, CA) and pAAV9 (Konno et al., 2014). We adopted adeno-associated virus packed with serotype 9 capsid (AAV-9) as a delivery vehicle because this vector has a strong tropism for cardiomyocytes. The viral particles were purified using ammonium sulfate precipitation and iodixanol continuous gradient centrifugation (Miyake et al., 2012). The genomic titer of the purified AAV9 vector as determined by real-time PCR was 2. 68 × 10^13^–5.44 × 10^13^ vector genomes (vg)/mL.

#### Mdivi-1 preparation

Mdivi-1 (3-(2,4-dichloro-5-methoxyphenyl)-2-sulfanyl-4(3H)-quinazolinone) was purchased from MedChemExpress (HY-15886) and dissolved in dimethylsulphoxide (DMSO; 100 mg/mL) as a stock solution. For mice injections, Mdivi-1 was diluted in sterile saline (1% DMSO). Mdivi-1 solution was gently sonicated (SONIFIER 150, BRANSON) at a power level 1 for 15 s and heated on 45°C for producing a homogenous suspension. Reagents were used immediately after preparation. For cell culture experiments, Mdivi-1 stock solution was diluted in culture medium to varying working concentrations.

#### Isoproterenol pump implantation and Mdivi-1 treatments

For all studies, 8- to 12-week-old male C57BL/6 or MITOL KO mice were randomly assigned to receive i.p. injections of either isoproterenol (60 mg/kg, #19703-04, nacalai tesque) or saline for 7 days using mini-osmotic pumps (model: alzet-20; Durect Corporation, CA. USA). Pumps were dorsally implanted in the mice for 7 days. Mice received once daily i.p. injections (50 mg/kg) with mdivi-1 beginning on the day of the first isoproterenol treatment and continued until mice were collected 7 days. At the end of the treatment period, pumps were surgically removed.

#### Blue Native PAGE

Blue-native PAGE (BN-PAGE) was performed according to Schagger (2001). Mitochondrial fraction isolated from heart tissues were solubilized with solubilization buffer (1% digitonin (#044-02121, WAKO), 20 mM NaCl, 50 mM imidazole (#091-00012, WAKO) pH 7.0, 1 mM EDTA, 10% glycerol, 500 mM 6-aminocaproic acid (#103200, WAKO), protease inhibitors (#11697498001, Roche)). After centrifugation at 14,000 × g for 15 min at 4°C Coomassie Brilliant blue G-250 (0.2% final concentration) was added, and the samples were electrophoresed through 3–12% polyacrylamide gradient gels. The gels were subjected to IB using the Abs described in the figures.

#### Immunoprecipitation

To analyze the interaction between endogenous MITOL and Drp1, crude mitochondria from mouse heart were solubilized with lysis buffer (0.5% NP-40, 50 mM Tris-HCl pH 7.4, 150 mM NaCl, 5 mM EDTA, 20% sucrose, protease inhibitors). Lysates were centrifuged at 15,000 rpm for 10 min at 4°C, and the supernatant was subjected to IP using an anti-MITOL Ab and normal rabbit IgG as a control. For the ubiquitination assay, cells transfected with vectors as indicated in the figures were lysed with 0.1% SDS RIPA buffer (0.1% SDS, 0.05% DOC, 1% TritonX-100, 10 mM Tris-HCl pH 7.4, 150 mM NaCl, 5 mM EDTA, protease inhibitors), and then incubated with the appropriate Ab.

#### Mitochondrial respiration

Mitochondria were prepared from mice (Frezza et al., 2007). Mitochondrial oxygen consumption with 5 mM succinate as a respiratory substrate was measured at 37°C using a Clark electrode (Rank Brothers, Cambridge, United Kingdom) calibrated with air-saturated respiration buffer comprising 0.115 M KCl, 10 mM KH2PO4, 3 mM HEPES (pH 7.2), 2 mM MgCl_2_, 1 mM EGTA and 0.3% (w/v) defatted BSA, assumed to contain 406 nmol atomic oxygen/mL (Reynafarje et al., 1985). Respiratory rates with 4 mM pyruvate + 1 mM malate as a substrate in State 3 (with 0.25 mM ADP) and State 4 (with 1 μg/mL oligomycin) were determined using the Oxygen Meter Model 781 and the Mitocell MT200 closed respiratory chamber (Strathkelvin Instruments). Basal respiration (state 2), phosphorylating respiration in the presence of ADP (state 3), resting respirations with oligomycin (state 4), and maximal uncoupling respiration in the presence of FCCP (uncoupled) were examined using optimal amount of heart mitochondria.

#### Morphological analysis by immunofluorescence microscopy

Cells were fixed with 4% paraformaldehyde in phosphate-buffered saline (PBS) for 30 min at room temperature, then washed twice with PBST, permeabilized with 0.2% Triton X-100 in PBS for 10 min, then washed 3 times with PBST, and blocked with 5% bovine serum albumin in PBST, all at room temperature. The cells were incubated with indicated primary antibodies for one hour at room temperature, washed three times with PBS, and then incubated with appropriate secondary antibodies for 30 min. The samples were washed as described above, mounted using Fluorescent Mounting Medium (S3023, Dako), and analyzed using an Olympus IX81 confocal fluorescence microscope and an Olympus FV1000 laser scanning microscope. Data are the mean ±SEM of 3 independent experiments.

#### Apoptosis assays

Apoptosis was determined by the terminal deoxyribonucleotidyl transferase-mediated TdT-mediated dUTP nick end labelling using a kit from Roche (#11684795910) according to the kit’s instructions.

#### Flow cytometric analyses of isolated mitochondria

Mitochondria isolated from the heart were stained with 200 nM MitoTracker Green (M7514, Invitrogen), 2.5 mM MitoSOX red (M36008, Invitrogen) at room temperature for 30 min and washed twice with PBS. Flow cytometric analyses of mitochondrial size (forward scatter, FSC), mitochondrial superoxide level (MitoSOX red signal intensity detected by PE channel) were performed on a Cell Sorter SH800 (SONY, Japan). Data are shown as histograms for, and as bar graph of average signal intensity of, 30,000 ungated events.

#### Isolation of cardiac mitochondria

Protocol for Mitochondrial isolation was developed from previously published protocols (Frezza et al., 2007). Mouse hearts were collected on ice, minced finely, and homogenized with a glass/teflon Potter Elvehjem homogenizer. Heart homogenates were centrifuged at 800 g × 10 min at 4°C and the supernatant collected and centrifuged at 8,000 g × 10 min at 4°C; both the pellet and the supernatant were collected. The pellet was again washed and centrifuged at 8,000 g × 10 min at 4°C to obtain cardiac mitochondria, which were resuspended for analyses and measurements were performed immediately following isolation.

#### Histological studies

Hematoxylin and eosin staining (#051-06515, #131-09665, WAKO), Masson’s trichrome staining (HT15-1KT, Sigma), TUNEL assay, and immunofluorescent staining were performed on sections from formalinfixed, paraffin-embedded tissues. The myocyte cross-sectional area was measured from images captured from wheat germ agglutinin (WGA, W11261, Invitrogen)-stained sections. The outlines of 100-300 myocytes were traced in each section using NIH ImageJ. Lipofuscin was detected by autofluorescence in fluorescence microscopy. Lipofuscins appears as irregular granules that emit yellow-orange fluorescence between 500 and 640 nm, under any excitation wavelength ranging from 360 to 647 nm. SA-β-gal activity was determined using the Senescence β-Galactosidase Staining Kit (#9860S, Cell Signaling Technology) according to the manufacturer’s instructions. For mouse hearts, β-gal staining solution was used at a final pH of 5.0.

#### MITOL-knockout mice

To generate inducible cardiomyocyte-specific MITOL knockout animals, mice bearing MITOL floxed alleles (MITOL^Flox/FLox^) were crossed with transgenic mice expressing MerCreMer under the control of the cardiac α-myosin heavy chain (αMHC) (Sohal et al., 2001). Tamoxifen-induced Cre LoxP recombination was activated by intraperitoneal administration of tamoxifen (#13258, Cayman Chemical) for 5 days. Cre-negative littermates, also receiving tamoxifen treatment, were used as controls. All animals were maintained under university guidelines for the care and use of animals. The experiments were performed after securing Tokyo University of Pharmacy and Life Sciences Animal Use Committee Protocol approval. PCR genotyping was performed with the following primers:

MITOL Fw: CACAGGTACGGTAGGTGTGTAAGC

MITOL Rv: ATGGGAATGTGGTTCAGTTGTACC

Cre Fw: GTTTCACTGGTTATGCGGCGG

Cre Rv: TTCCAGGGCGCGAGTTGATAG

#### Transmission electron microscopy

Hearts were quickly removed from the chest after euthanasia. The heart was fixed with 1% glutaraldehyde/4% PFA in 0.1 M sodium cacodylate buffer, pH 7.4 for 24 h. Small tissue blocks (1–2 mm^3^) were then excised from left ventricular lateral mid-walls. Thin sections were observed at 5,000–15,000 × magnification (BML, inc. Japan). Mitochondrial content was measured as the areas taken by mitochondria compared to those of the cardiomyocytes using ImageJ.

#### Mitochondrial ROS production

Mitochondrial ROS were examined using MitoSOX Red. Cells were incubated with MitoSOX (5 μM) for 10 min at 37°C, washed with PBS, changed to phenol red-free DMEM, and imaged by confocal microscopy (Olympus).

#### Detection of carbonylated proteins

Protein carbonyl content was measured with a detection kit (cat. #ROIK03; SHIMA Laboratories, Tokyo, Japan). Samples were prepared as described above. The membrane after the transfer was reacted with 2,4-dinitrophenylhydrazine, and the protein-bound 2,4-dinitrophenylhydrazone was detected with an anti-dinitrophenyl (DNP) antibody.

#### Measurement of cardiac ATP levels

Hearts were collected and homogenized in ice-cold homogenization solution. Cardiac mitochondrial ATP levels were measured using a luciferase-based ATP determination kit (TA100, TOYO B NET, Japan).

#### Transverse aortic constriction (TAC) surgery

TAC-surgery was performed to create left ventricular pressure overload. Control and tamoxifen-treated mice (weighing 20–22 g) were anesthetized using 100 mg/kg ketamine and 5 mg/kg xylazine. After orotracheal intubation, the cannula was connected to a volume-cycled ventilator (SN-480-7, Shimano, Japan) using room air at a tidal volume of 0.3 mL and a respiratory rate of 140 breaths/min. A small incision through the second intercostal space was made to enter the chest cavity and the transverse aorta was constricted with 7-0 nylon string by ligating the aorta with a blunted 27-gauge needle, which was later removed.

#### Echocardiography

Transthoracic echocardiography was performed to evaluate cardiac function using the Aplio 300 (Toshiba Medical System) and a 14-MHz transducer. Mice were initially anesthetized with 2% isoflurane, reduced to 1% during examinations. Left ventricular short-axis dimensions at the tip of the papillary muscles were measured on M-mode. Fractional shortening was calculated as (LVDd - LVDs)/LVDd × 100 (%).

#### Isolation of adult mouse ventricular cardiomyocytes

Cardiomyocytes were isolated from left ventricular tissue using a method similar to Shioya (2007). Hearts were rapidly removed from mice anesthetized with an overdose of pentobarbital (300 mg/kg, i.p.). Isolated hearts were perfused with cell-isolation buffer (CIB; 130 mM NaCl, 5.4 mM KCl, 0.5 mM MgCl_2_, 0.33 mM NaH_2_PO_4_, 22 mM glucose, 50 nM/mL bovine insulin (I6634, Sigma) and 25 HEPES-NaOH (pH 7.4)) supplemented with 0.4 mM EGTA at 37°C. After 3-4 min, the perfusate was changed to the enzyme solution, which was CIB containing 0.3 mM CaCl_2_, 1 mg/mL collagenase (CLS-2, Worthington Biochemical), 0.06 mg/mL protease (P5147, Sigma) and 0.06 mg/mL trypsin (T8003, Sigma). After 6-9 mi, the tissue was cut into several pieces and further digested in fresh enzyme solution containing 0.7 mM CaCl_2_ and 2 mg/mL BSA (A9418, Sigma) for 15–20 min at 37°C. After the cell suspension was centrifuged at 14 × *g* for 3 min, the cell pellet was resuspended in CIB containing 1.2 mM CaCl_2_ and 2 mg/mL BSA, and then incubated for 10 min at 37°C. The cell suspension was centrifuged (14 × *g*, 3 min) again and resuspended in Tyrode’s solution (140 mM NaCl, 5.4 mM KCl, 1.8 mM CaCl_2_, 0.5 mM MgCl_2_, 0.33 mM NaH_2_PO_4_, 11 mM glucose, 2 mg/mL BSA and 5 mM HEPES-NaOH (pH 7.4). Experiments of isolated cardiomyocytes were performed with cells superfused with Tyrode’s solution unless otherwise indicated.

#### Measurement of cell shortening

Isolated cardiomyocytes were electrically stimulated at 0.5, 1, 2, 4 Hz using a two-platinum electrode electrode insert connected to a bipolar stimulator (Nihon Kohden, SEN-3301). Cell images were recorded at a time resolution of 20 ms with a High-performance EvolveTM EMCCD camera (Photometrics) coupled to an inverted microscope (IX71, Olympus) with a 20× water immersion objective lens (UApo N340, Olympus) and analysed using MetaMorph software (version 7.7.1.0; Molecular Devices).

#### Measurement of intracellular Ca^2+^ transients in adult cardiac myocytes

Intracellular Ca^2+^ transients were measured using Indo-1 AM. Isolated cardiomyocytes were loaded with 10 μmol/L Indo-1 AM (Dojindo) for 10 min at 37°C and electrically stimulated at 1 Hz. Ca^2+^ transients were measured as the ratio of fluorescence emitted at 405/480 nm after excitation at 340 nm. Ca^2+^ transients were recorded under an inverted IX71 microscope with a 20× water immersion objective lens (UApo N340) and a High-performance EvolveTM EMCCD camera and analysed using MetaMorph software.

#### Measurement of SR Ca^2+^ content

SR Ca^2+^ content was measured with Fura-2 AM and a caffeine spritz. Isolated cardiomyocytes were loaded with 2 μM Fura-2 AM (I006, Dojindo) for 30 min at 37°C. Myocytes were excited at 340 and 380 nm using a Lambda DG-4 Ultra High Speed Wavelength Switcher (Sutter Instruments) coupled to an inverted IX71 microscope with a UApo 20×/0.75 objective lens (Olympus). Before the rapid application of 10 mM caffeine in Tyrode’s solution, myocytes were electrically stimulated at 0.2 Hz to obtain steady-state contractions. Peak amplitude (ΔF340/F380) of caffeine-induced Fura-2 transient was taken as an index of SR Ca^2+^ content. Fura-2 fluorescent signals were recorded with ORCA-Flash 2.8 (Hamamatsu Photonics) and analysed by a ratiometric fluorescence method using MetaFluor software (version 7.7.5.0; Molecular Devices).

### Quantification and statistical analysis

Data are expressed as mean s.e.m. Statistical analysis was performed with GraphPad PRISM version 8 (GraphPad Software, Inc.). Data groups (two groups) with normal distribution were compared using two-sided unpaired Student’s *t-*test. The Mann-Whitney *U*-test was used for nonparametric data. Comparisons between multiple groups were assessed by one-way or two-way ANOVA with Bonferroni *post hoc* analysis. ∗p < 0.05; ∗∗p < 0.01; NS, not significant. No statistical method was used to predetermine sample size.

## Data Availability

• Data: All data reported will be shared by the lead contact upon request. • Code: This paper does not report original code. • Additional information required to analyze the data reported in this paper is available from the lead contact upon request.

## Supplemental information

Document S1. Figures S1–S6

Table S1. The qPCR primer sequences related to STAR Methods
