# Supplementary material for: Protective roles of MITOL against myocardial senescence and ischemic injury partly via Drp1 regulation
Source: iScience. 2022 Jun 11;25(7):104582. doi: 10.1016/j.isci.2022.104582 (PMC9249672; doi:10.1016/j.isci.2022.104582)
Supplement: Document S1. Figures S1–S6 [file mmc1.pdf]

iScience, Volume *25*

Supplemental information

Protective roles of MITOL against myocardial senescence and ischemic injury partly via Drp1 regulation

Takeshi Tokuyama, Hideki Uosaki, Ayumu Sugiura, Gen Nishitai, Keisuke Takeda, Shun Nagashima, Isshin Shiiba, Naoki Ito, Taku Amo, Satoshi Mohri, Akiyuki Nishimura, Motohiro Nishida, Ayumu Konno, Hirokazu Hirai, Satoshi Ishido, Takahiro Yoshizawa, Takayuki Shindo, Shingo Takada, Shintaro Kinugawa, Ryoko Inatome, and Shigeru Yanagi

Supplementary Figure 1

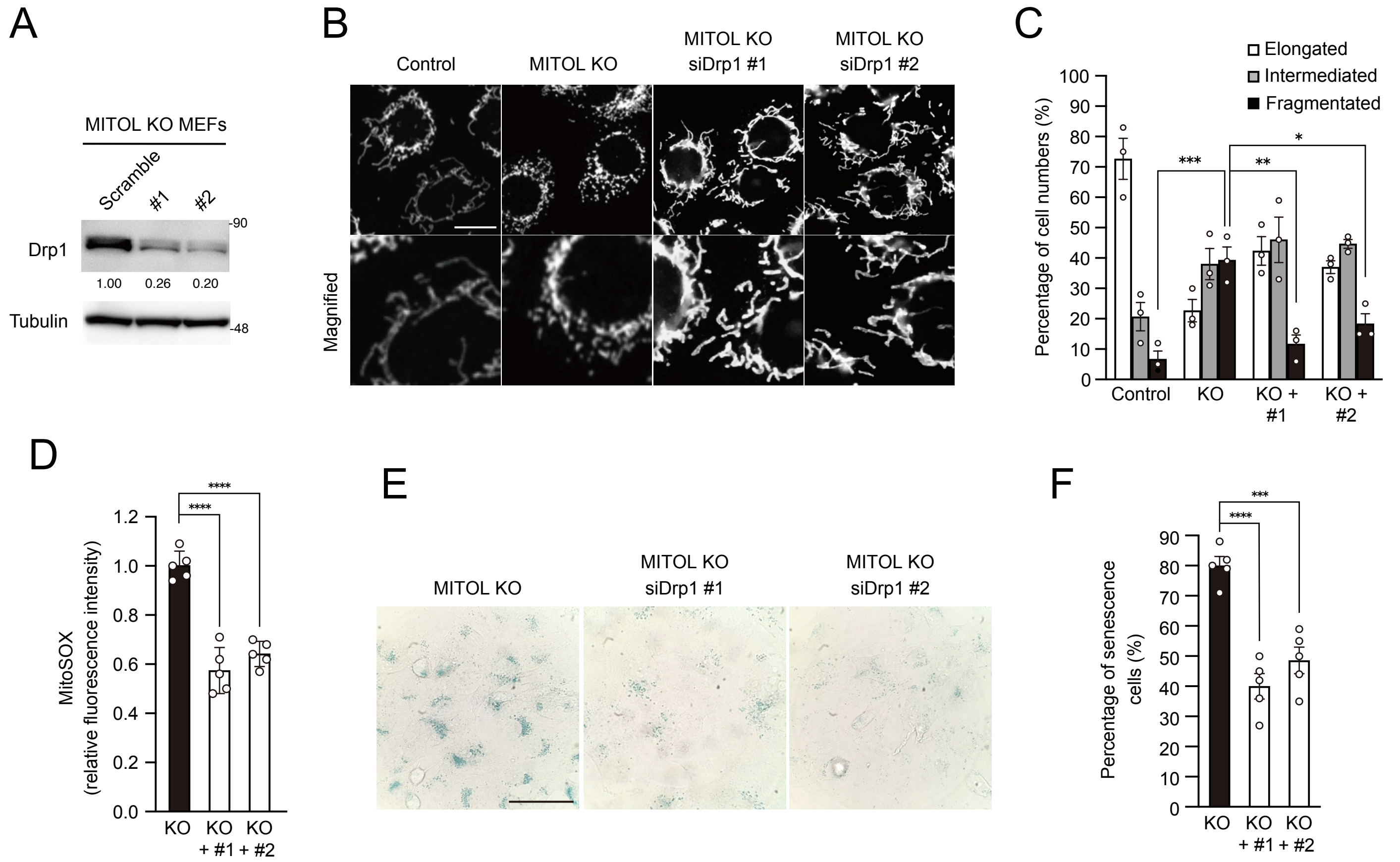

Supplemental figure 1 related to Figure 1. Drp1 knockdown partially rescued cellular senescence in MITOL KO MEFs.

(A) Knockdown of Drp1 in MITOL-KO MEFs. MITOL-KO MEFs were transfected with scramble or siRNA against Drp1 (#1 and #2) for 24 hours and then lysates ware immunoblotted with anti-Drp1 antibody.

(B and C) Mitochondrial fragmentation in MITOL-KO MEFs. MITOL-KO MEFs transfected with scramble or siRNA against Drp1 (#1 and #2) for 24 hours were stained with MitoTracker and mitochondrial morphologies were compared (B). Bar, 10 μ m. The bottom panels show high-magnification images of the boxed regions. Percentages of cells showing each mitochondrial morphology were calculated (C). Mean, ±SEM (n=3). Analysis was performed with two-way ANOVA followed by Bonferroni post hoc analysis. *P < 0.05, **P < 0.01, ***P < 0.001.

(D) Mitochondrial ROS is upregulated in MITOL-KO MEFs. MITOL-KO MEFs transfected with scramble or siRNA against Drp1 (#1 and #2) for 24 hours were stained with MitoSOX and mitochondrial-derived superoxide generation was measured by flowcytometric analysis. Bar graphs show relative levels of mean fluorescence intensity of MitoSOX compared with the intensity of control MEFs. Error bars indicate, ±SEM (n=5). Analysis was performed with one-way ANOVA followed by Bonferroni post hoc analysis. ****P<0.0001.

(E and F) Drp1 knockdown attenuated cellular senescence in MITOL KO MEFs. Cytochemical staining of SA-β-gal activity in MEFs transfected with scramble or siRNA against Drp1 (#1 and #2) for 24 hours. Bar, 50 μm. The bar graph shows the percentages of SA-β-gal positive cells calculated from 100 cells of each MEFs shown in E. Mean ±SEM (n=5). Analysis was performed with one-way ANOVA followed by Bonferroni post hoc analysis. ***P<0.001, ****P<0.0001 (F).

Supplementary Figure 2

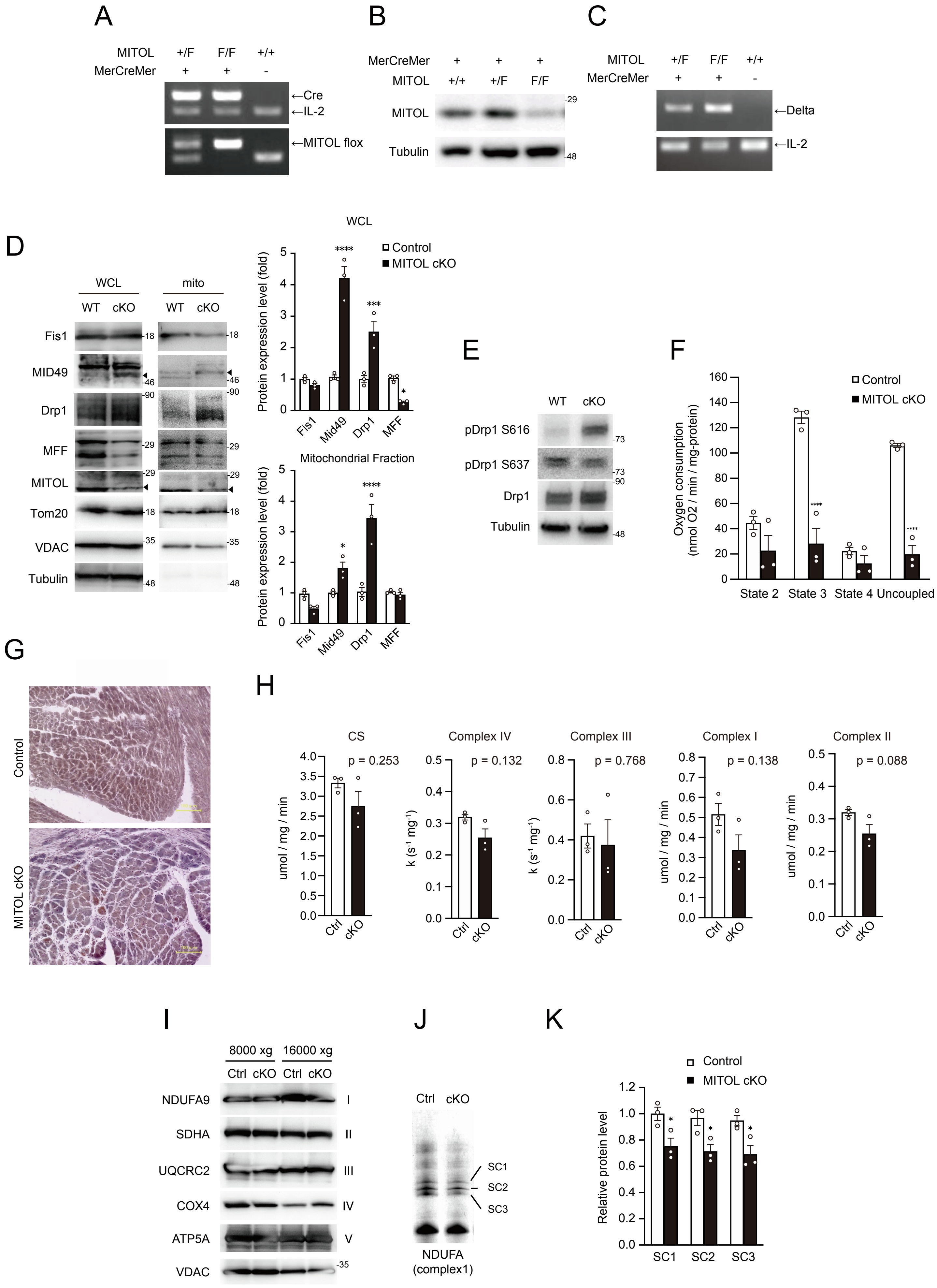

Supplemental figure 2 related to Figure 2. Generation and characterization of heart-specific MITOL-KO mice.
(A) αMHC-MerCreMer mice were crossed with MITOLF/F mice. Mice were genotyped using specific PCR primers to detect the genomic locus of Cre, IL2, MITOLWT, and MITOLflox.
(B and C) Protein and mRNA expression levels of MITOL in hearts were confirmed by immunoblotting (B) and RT-PCR (C).
(D) Protein expression levels of MITOL substrates in the heart. Mitochondrial fractions were isolated from cardiomyocytes six months after tamoxifen treatment, followed by immunoblotting with indicated antibodies. The relative protein levels were quantified by densitometry. Data are standardized to tubulin (WCL) or VDAC (mitochondrial fraction) levels, and are expressed relative to control mice. Mean ±SEM (n=3). Analysis was performed with two-way ANOVA followed by Bonferroni post hoc analysis. ****P < 0.0001, ***P < 0.001, *P < 0.05.
(E) Immunoblotting assay was performed on cardiac lysates from control and cKO mice six months after tamoxifen treatment with indicated antibodies.
(F) Reduced oxygen consumption rate of mitochondria isolated from MITOL-KO mouse cardiomyocytes. State 3 and State 4 respiration rate of mitochondria with pyruvate/malate as a respiratory substrate. The maximum respiration rate was caused by addition of an uncoupler CCCP. Mean ±SEM (n=3). Analysis was performed with two-way ANOVA followed by Bonferroni post hoc analysis. ****P < 0.0001.
(G) Reduced respiratory chain activity in MITOL-KO mice. COX-SDH double staining was performed on frozen heart sections in cKO mice six months after tamoxifen-treatment. Scale bars, 100 μm.
(H) The activities of each respiratory chain complex (RCC) were comparable between control and MITOL-KO mitochondria. Cardiac mitochondria of control and MITOL-KO mice were isolated and activities of each RCC were measured. Error bars indicate ±SEM (n=3).
(I) Immunoblot analysis for each electron transport chain complex I-V, respectively.
Each mitochondrial fraction was isolated after centrifugation at 8000g or 16000g from heart six months after tamoxifen treatment, followed by immunoblotting with indicated antibodies.
(J and K) Impaired respiratory chain supercomplex (SC) formation in MITOL-KO mitochondria. Immunoblot of assembled supercomplexes in digitonin-permeabilized mitochondria separated by BNGE and probed with monoclonal antibodies for CI (anti-NDUFA9). BN-PAGE assay was performed to detect SC in cardiac mitochondria (J). The relative levels of SC were quantified by densitometry and are compared relative to control mice (K). Mean ±SEM, n=3, *P <0.05; Student’ s t-test.

Supplementary Figure 3

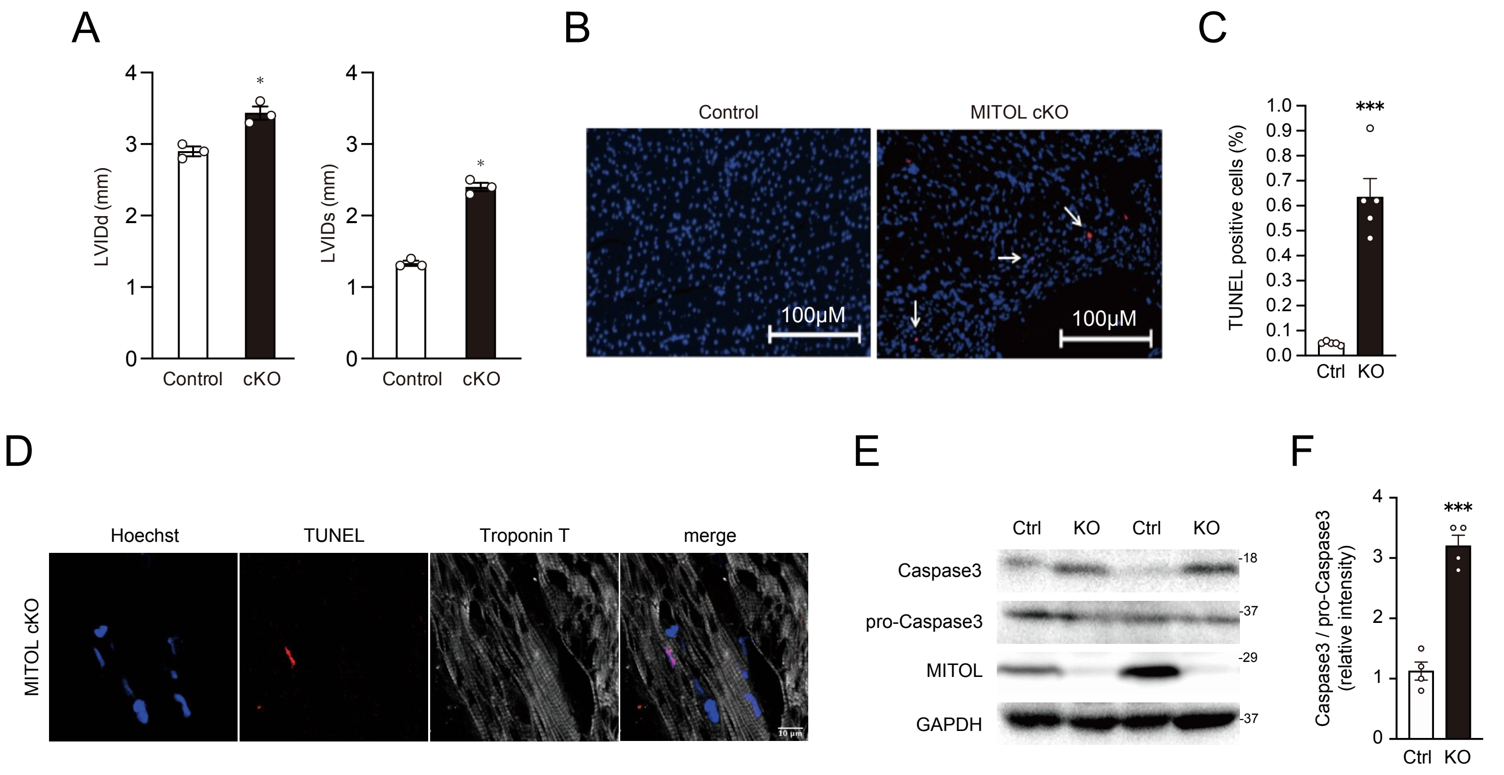

Supplemental figure 3 related to Figure 3. Enhanced cell death by MITOL deletion.
(A) Cardiac dysfunction in MITOL-cKO mice six months after tamoxifen-treatment. Echocardiographic analysis of left ventricular dimensions and cardiac function in mice. LVIDd; diastolic left ventricular internal diameters. LVIDs; systolic left ventricular internal diameters. Mean ±SEM (n=3). *P<0.05.
(B and C) Increased TUNEL-positive cells in MITOL-cKO heart. Representative views of cross-sections of control and cKO mouse heart six months after tamoxifen-treatment stained with TUNEL and DAPI to detect apoptotic cells (B). Bars, 100 μm. The percentages of TUNEL-positive cells were measured (C). Mean ±SEM (n=5). ***P<0.001; Student's t-test.
(D) TUNEL positive cardiomyocyte in MITOL-cKO. Representative image of control and cKO mouse heart six months after tamoxifen-treatment. Cardiomyocytes were immunostained with Troponin T. Bars, 10 μm.
(E and F) Cleaved caspase-3, a marker of apoptosis, was upregulated in cKO cardiomyocytes. Immunoblotting assay was performed on the lysate of cardiomyocytes from control and cKO mice six months after tamoxifen-treatment with anti-caspase-3 and anti-cleaved caspase-3 antibodies. Bar graph shows the ratio of cleaved caspase to total caspase. Mean ±SEM (n=4). ***P<0.001; Student's t-test.

Supplementary Figure 4

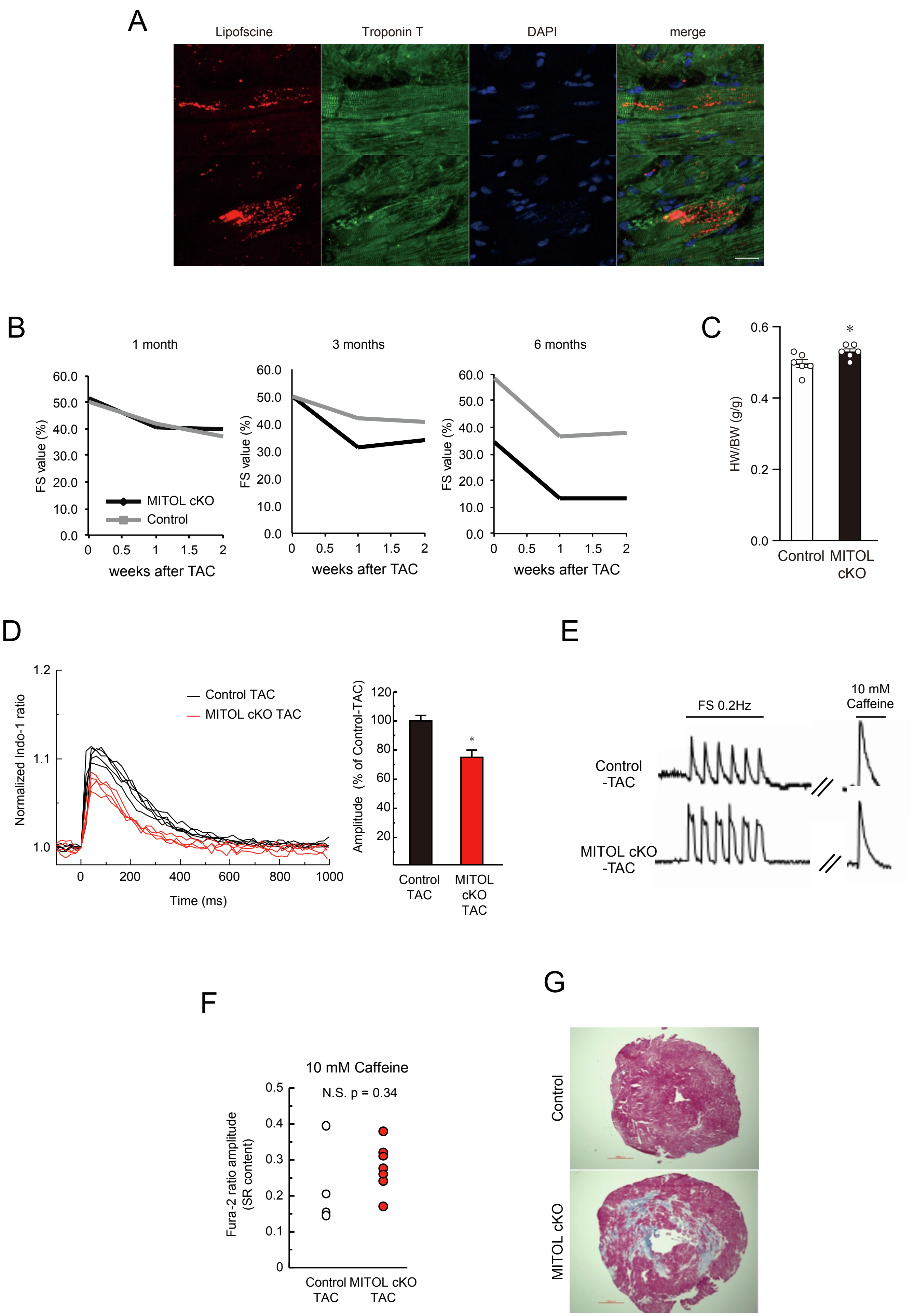

Supplemental figure 4 related to Figure 4. Contractility and Ca2+ handling of single cardiomyocytes isolated from control-TAC and MITOL-KO-TAC mice.

(A) Lipofuscin deposition in cardiomyocytes. Troponin T staining was performed on frozen heart sections in cKO mice six months after tamoxifen-treatment. Lipofuscin was detected as autofluorescence. Scale bars, 20 μm. All images are from MITOL cKOs.

(B) Impaired systolic function in MITOL-KO-TAC mice. MITOLflox/flox mice, and MITOLflox/flox;αMHC-CreMer mice one, three, six months post tamoxifen treatment were subjected to TAC surgery. Systolic function was monitored one or two weeks after TAC surgery. Graphs show the percentage of FS (n=3). FS, fractional shortening of left ventricular diameter.

(C) Heart weight to body weight ratio of mice. Heart-to-body weight ratio in control-TAC and MITOL-KO-TAC mice. Mean ±SEM (n=5). *P<0.05, Student' s t-test.

(D) Intracellular Ca2+ transients were measured using Indo-1 AM. Peak amplitude of Ca2+ transient. Data were normalized to control-TAC mice. *p < 0.05 compared with control-TAC mice.

(E) Ca2+ transients obtained by measuring the mean cellular fluorescence from six consecutive Ca2+ transients. SR calcium content was estimated using rapid application of caffeine.

(F) SR Ca2+ content estimated from peak amplitude of caffeine-induced Ca2+ transient. Mean ±SEM (Control-TAC=5, MITOL-cKO-TAC=7). Data were normalized to control-TAC mice. *p < 0.05 compared with control-TAC mice.

(G) Myocardial fibrosis in control-TAC and MITOL-KO-TAC mice. Representative photographs show Masson' s trichrome staining for collagen.

Supplementary Figure 5

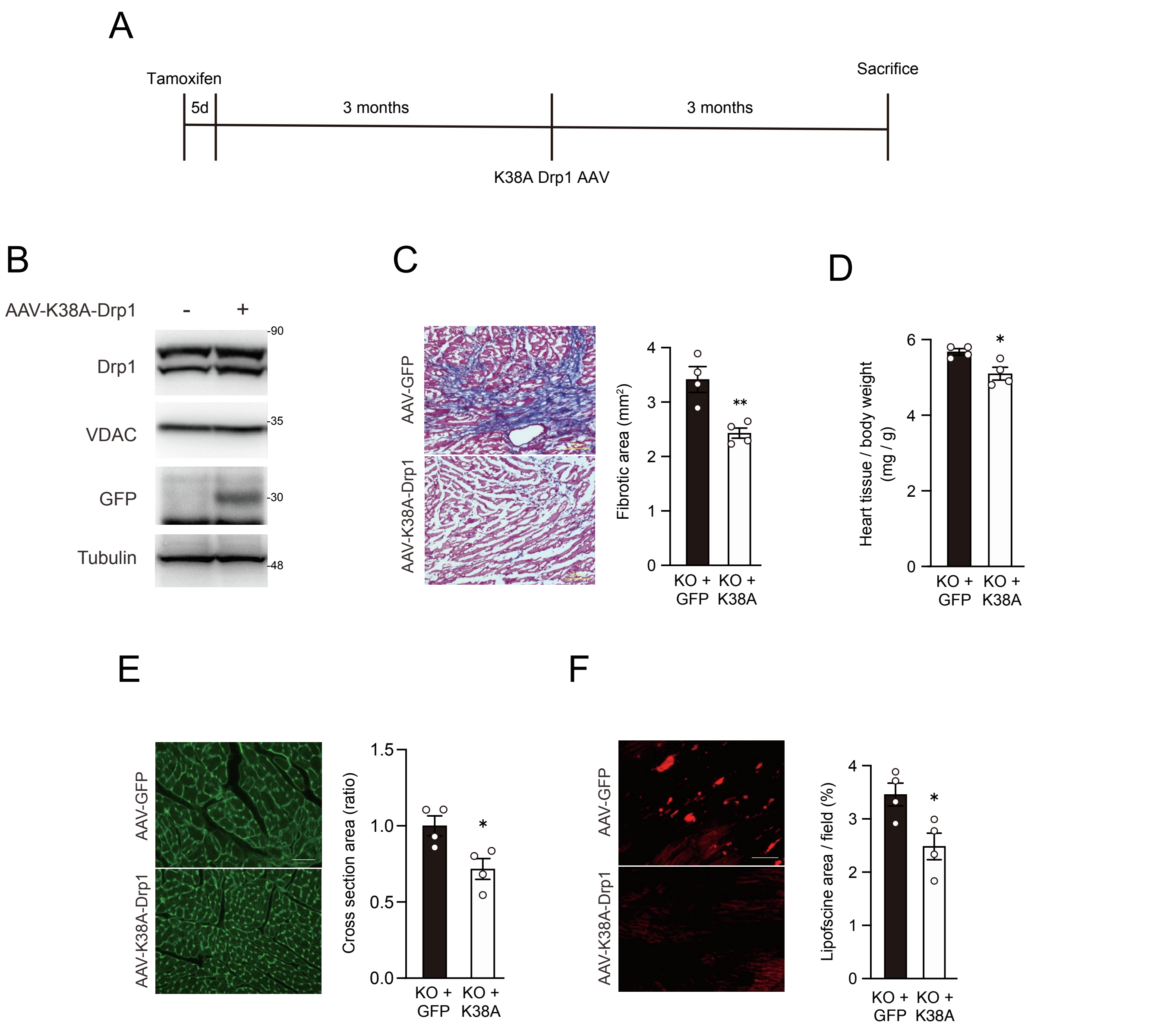

Supplemental figure 5 related to Figure 5. Delivery of dominant negative Drp1 into cardiomyocytes decreased cell death by MITOL deletion.

(A) Study design for the transduction of K38A-Drp1 in MITOL cKO mice. Three months after MITOL knockout, mice were introduced with AAV vectors driven by a cardiac cTNT promoter (GFP: AAV-GFP and GFP-P2A-K38A-Drp1: AAV-K38A-Drp1) packaged in AAV-9 capsides via intrathoracic injection. Three months after virus injection, hearts were dissected and the expression of GFP and Drp1 was assessed by immunoblotting.

(B) Drp1 was upregulated in cardiomyocytes of MITOL cKO mice transduced with AAV-K38A-Drp1. IB assay of Drp1, GFP and tubulin was performed on lysates of total mice heart cells. Data are standardized to tubulin levels and are expressed relative to AAV-GFP injected mice.

(C) Myocardial fibrosis in cKO mice was attenuated by AAV-K38A-Drp1. Representative photographs show Masson’ s trichrome staining for collagen. Fibrotic areas were measured. Mean ±SEM (n=4). **$P < 0.01$.

(D) AAV-K38A-Drp1 delivery blocked the increase in heart weight of MITOL-deficient hearts. Heart-to-body weight ratio in cKO mice as described in (A) was shown, respectively. Mean ±SEM (n=4). Analysis was performed with two-way ANOVA followed by Bonferroni post hoc analysis. *$P < 0.05$.

(E) AAV-K38A-Drp1 delivery blocked cardiomyocyte hypertrophy. Frozen heart tissue sections were stained with FITC-conjugated wheat germ agglutinin (WGA) to detect cardiomyocyte borders. The ratio of cardiomyocyte area was measured from 100 cells. Mean ±SEM (n=4). *$P < 0.05$. Bar, 50 μm.

(F) AAV-K38A-Drp1 delivery reduced the senescent marker lipofuscin. Frozen heart tissue sections were analyzed for lipofuscin by fluorescence microscopy. Relative content of lipofuscin (area of lipofuscin granules/field of the myocardium) was analyzed with Image J. Mean ±SEM (n=4). *$P < 0.05$. Bar, 20 μm.

Supplementary Figure 6

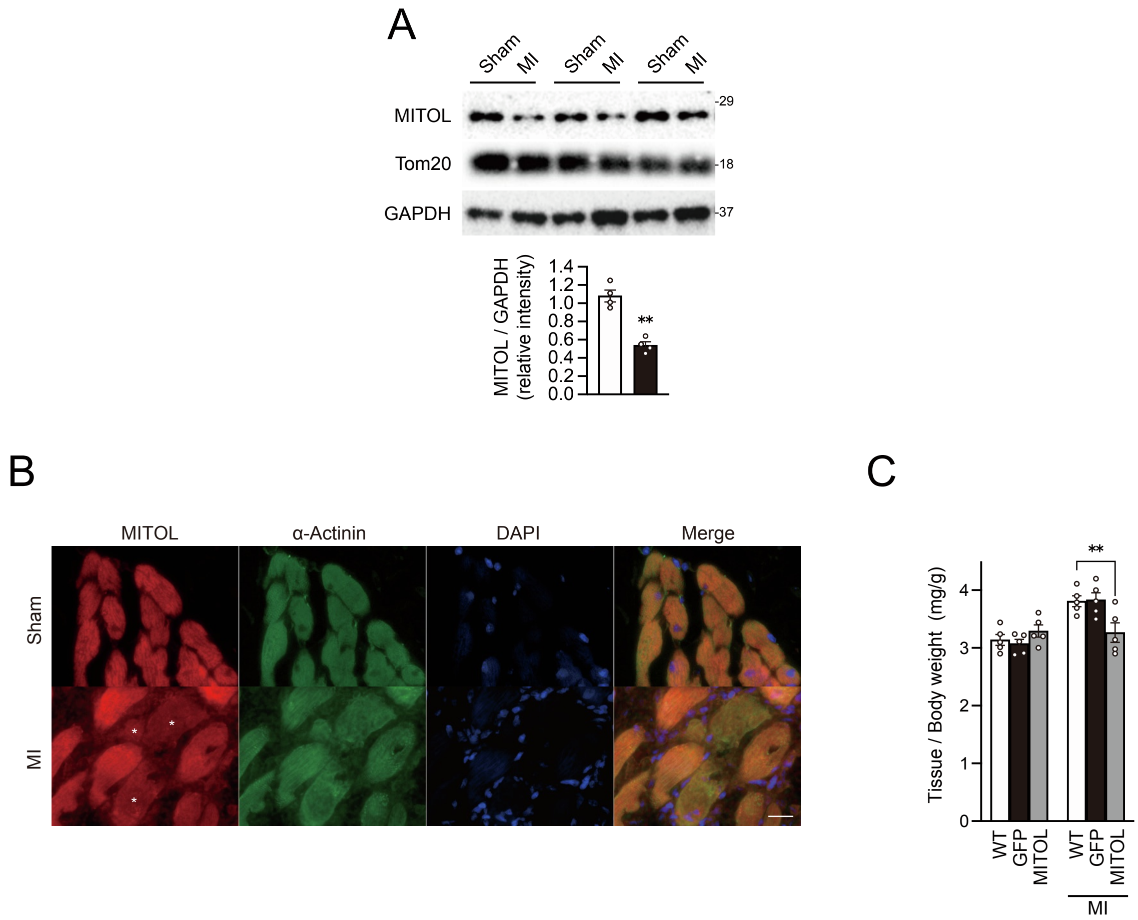

Supplemental figure 6 related to Figure 6. Downregulation of MITOL in ischemia-induced rodent heart.
(A) Immunoblot analysis indicates downregulation of MITOL in infarct mouse cardiomyocytes. Myocardial infarction (MI) was induced by ligation of left coronary artery in mice. After 4 weeks of MI, IB assay of MITOL and Drp1 was performed on whole lysates isolated from sham and MI-induced mouse hearts. Anti-Tom20 and anti-GAPDH antibodies were used as a mitochondrial marker and a cytosolic marker, respectively. Data are standardized to GAPDH levels and are expressed relative to sham operated mice. Mean ±SEM (n=4). **P < 0.01, Student' s t-test.
(B) Immunofluorescent analysis indicates downregulation of MITOL in infarct rat cardiomyocytes. Antibodies against MITOL and actinin were applied to frozen rat heart sections after sham surgery or MI operation. Asterisks indicate cardiomyocytes that show decreased expression of MITOL. Scale bars, 20 μm.
(C) Heart weight to body weight ratio of mice. Error bars indicate ±SEM (n=5). Analysis was performed with two-way ANOVA followed by Bonferroni post hoc analysis. **P < 0.01.
